# Variation of a major facilitator superfamily gene contributes to differential cadmium accumulation between rice subspecies

**DOI:** 10.1038/s41467-019-10544-y

**Published:** 2019-06-12

**Authors:** Huili Yan, Wenxiu Xu, Jianyin Xie, Yiwei Gao, Lulu Wu, Liang Sun, Lu Feng, Xu Chen, Tian Zhang, Changhua Dai, Ting Li, Xiuni Lin, Zhanying Zhang, Xueqiang Wang, Fengmei Li, Xiaoyang Zhu, Jinjie Li, Zichao Li, Caiyan Chen, Mi Ma, Hongliang Zhang, Zhenyan He

**Affiliations:** 1 Key Laboratory of Plant Resources, Institute of Botany, Chinese Academy of Sciences, Beijing, 100093 China; 2 Key Lab of Crop Heterosis and Utilization of Ministry of Education, Beijing Key Lab of Crop Genetic Improvement, China Agricultural University, Beijing, 100193 China; 3 College of Biological Sciences and Biotechnology, Beijing Forestry University, Beijing, 100083 China; 4 University of Chinese Academy of Sciences, Beijing, 100049 China; 5 Key Laboratory of Agro-Ecological Processes in Subtropical Region, Institute of Subtropical Agriculture, Chinese Academy of Sciences, Changsha, 410125 China

**Keywords:** Plant breeding, Abiotic, Agricultural genetics, Genome-wide association studies

## Abstract

Cadmium (Cd) accumulation in rice grain poses a serious threat to human health. While several transport systems have been reported, the complexity of rice Cd transport and accumulation indicates the necessity of identifying additional genes, especially those that are responsible for Cd accumulation divergence between *indica* and *japonica* rice subspecies. Here, we show that a gene, *OsCd1*, belonging to the major facilitator superfamily is involved in root Cd uptake and contributes to grain accumulation in rice. Natural variation in *OsCd1* with a missense mutation Val449Asp is responsible for the divergence of rice grain Cd accumulation between *indica* and *japonica*. Near-isogenic line tests confirm that the *indica* variety carrying the *japonica* allele *OsCd1*^*V449*^ can reduce the grain Cd accumulation. Thus, the *japonica* allele *OsCd1*^*V449*^ may be useful for reducing grain Cd accumulation of *indica* rice cultivars through breeding.

## Abstract (web summary)

Grain of *indica* rice accumulates more toxic cadmium (Cd) than *japonica*, but the underlying genetic basis is unclear. Here, the authors show that natural variation of *OsCd1* contributes to divergence in grain Cd accumulation and transferring *japonica* allele to *indica* rice leads to reduced Cd accumulation.

## Introduction

Cadmium (Cd) is a toxic heavy metal and can lead to Cd-related diseases such as renal tubular dysfunction and bone disease. Rice is a major component of diet for over half of the world's population. The accumulation of Cd is a serious threat to human being since it can be concentrated in body through the food chain^1^ and the biological half-life is estimated to be nearly 30 years^2^. Molecular genetic tools have been urgently sought to develop low-Cd rice to reduce potential health risks.

Generally, Cd is first absorbed from soil by roots, and then translocated into shoots, and accumulates into grains in final^3^. Transport systems, especially the genetic components locating on the membrane, play crucial roles in the Cd accumulation processes in rice. In root exodermis and endodermis, OsNramp1^4,5^ and OsNramp5^6–11^, which locate on the plasma membrane, are transporters for Cd uptake. The heavy metal ATPase, OsHMA3^12–15^, functions in vacuolar sequestration of Cd. In root stele, OsHMA2^16–18^, an efflux-type metal transporter on the plasma membrane, is suggested to facilitate Cd loading into the xylem. CAL1, a defensin-like protein preferentially expressed in root exodermis and xylem parenchyma cells, drives long-distance Cd transport via xylem vessels^19^. And in shoot node, OsLCT1^20,21^, a plasma membrane-localized efflux transporter, is responsible for Cd intervascular transfer.

By over-expression of the functional OsHMA3 or the disruption of OsLCT1 and OsHMA2, the grain Cd content can be reduced to some extent, which gives a clue that rice grain Cd contents can be reduced through regulating transporters expression. However, the rice Cd transport mechanism is complex and grain Cd accumulation is a result of cooperative interactions among multiple cells and issues. It is, therefore, worthwhile to explore more genetic loci involving in Cd accumulation of rice grain.

Rice varieties exhibit substantial genetic variation with respect to Cd accumulation ability^22–24^, which is a valuable resource for dissecting functional alleles and genetic improvement. OsHMA3 shows different Cd transport ability^13^ and OsNRAMP1 expresses in a different level among two cultivars^4^. By using chromosome segment substitution lines (CSSLs) carrying segments of *indica* rice Kasalath in a background of the *japonica* rice Koshihikari, several CSSLs have significantly lower Cd concentrations than that of Koshihikari^25^. However, current understanding of the genetic basis of rice Cd accumulation diversity remains at the level of the identification of several quantitative trait loci (QTLs)^25–27^. The natural allelic variations responsible for rice varietal differences have not been fully explored and the genetic basis of grain Cd accumulation differences remains unknown.

More recently, genome-wide association studies (GWAS) have been successfully used for identifying genes and alleles underlying several agronomic traits^28–32^, while the association study of rice grain Cd accumulation has not been attempted. Here, we show that a gene belonging to major facilitator superfamily, *OsCd1*, is associated with divergence in rice grain Cd accumulation. *OsCd1* locates at the plasma membrane of root and is involved in the grain Cd accumulation. Additionally, a single-nucleotide mutation, SNP22, diverges between *indica* and *japonica* and alters Cd transport ability of *OsCd1*. Interestingly, the natural variation *OsCd1*^*V449*^ in *japonica*, which is associated with a reduced Cd transport ability and decreased grain Cd accumulation, shows a potential value in low-Cd rice selection.

## Results

### *OsCd1* is associated with grain cadmium accumulation in rice

**Fig. 1:**
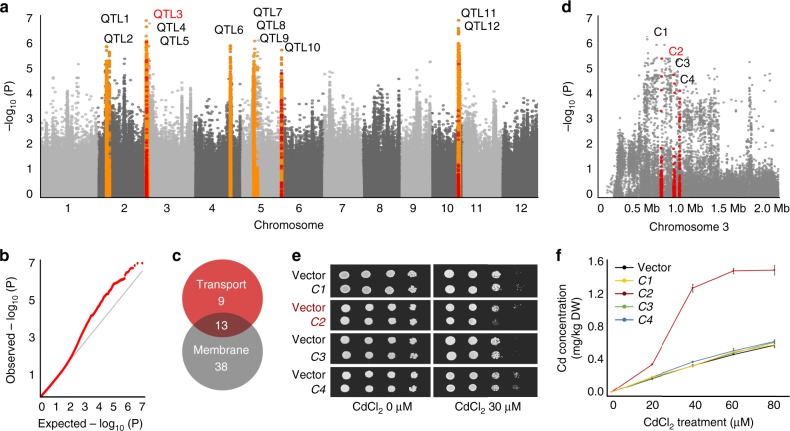
GWAS of the grain Cd accumulation. **a** Manhattan plots for grain Cd accumulation in diverse population. The orange pots indicated the 12 QTLs were identified as significantly associated with grain Cd accumulation. The red dots indicated the 14 candidate genes associated both with transport and membrane in GO Slim annotation. **b** The QQ plots for GWAS MLM model. **c** The numbers of genes annotated with transport and membrane. **d** The genome-wide association signals for grain Cd accumulation were shown in the region of 0–2 Mb on chromosome 3 (*x*-axis). The locations of four candidate genes *C1* (*LOC_Os03g02150*), *C2* (*LOC_Os03g02380*), *C3* (*LOC_Os03g02390*) and *C4* (*LOC_Os03g0248*0) were indicated with red pot respectively. **e** Dilution-series spot assays of yeast treated with and without 30 μM CdCl_2_. **f** Cd accumulation in wild-type W303 (black) and W303 expressing *C1* (yellow), *C2* (red), *C3* (green) and *C4* (blue) treated with CdCl_2_ for 24 h. Error bars indicate standard deviation. Data from the transgenic lines were designed with three replications and data points for all biological replicates are shown. Source data of Fig. 1f are provided as a Source Data file

In this study, we combined data from GWAS, gene annotation in GO Slim database and the yeast assay to look for genes associated with rice grain Cd accumulation. A set of 127 rice cultivars including 41 *japonica* and 86 *indica* from mini-core collection of rice in China^33^ and other wide-spread regions was used for GWAS of grain Cd concentration (Supplementary Fig. 1a, b). After testing rice varieties with a Cd treatment assay, we found that *indica* varieties could be phenotypically distinguished from *japonica* varieties by their significantly higher grain Cd accumulation (Supplementary Fig. 1c and Supplementary Data 1). Using 3,291,150 single-nucleotide polymorphisms (SNPs) with a minor allele frequency (MAF) > 0.05 covering the whole rice genome, we performed GWAS to identify the genetic loci associating with grain Cd accumulation. Under the compressed general mixed linear model (*P* < 1 × 10^−5^, MLM, threshold derived from 1000 permutation test) (Fig. 1a, b), 12 QTLs were identified as significantly associated with grain Cd accumulation (Supplementary Table 1). According to the rice genome annotation project website (MSU-RGAP), 494 genes were annotated to be located in the candidate locus (Supplementary Data 2). To identify the membrane transporter conferring Cd accumulation in rice, we then performed the gene ontology (GO) Slim analysis to select candidate genes associated with the keyword membrane and transport for further research. Altogether, 13 candidate genes located on chromosome 2, 3, 4, 6, and 10 were found to be associated both with transport and membrane in GO Slim annotation (Fig. 1c and Supplementary Data 3). Four candidate genes were selected on QTL3 of chromosome 3, which explained ~20.7% of the phenotypic variation (Fig. 1d), and transformed into *S. cerevisiae* to evaluate whether these candidate genes were involved in Cd transport.

Spot assays were tested with and without 30 μM CdCl_2_ and the growth rate was normalized by non-transgenic strains. As a result, only yeast transformed *C2* (*LOC_Os03g02380*; GenBank accessions AP014959.1) displayed sensitivity to Cd (Fig. 1e). Under 24 h Cd exposure, expression of *C2* enhanced Cd accumulation in a Cd dose-dependent manner while the other three showed no significant difference with the control (Fig. 1f). These results indicated that *C2* was the most likely candidate transporter gene in QTL3 and it was christened *OsCd1*.

*OsCd1* encoded a protein belonging to the MFS with 12 bilayer-spanning domains and the signature motif of MFS, G(93)slaD(97)kqG(100)rkR(103)^17^, located in the second cytoplasmic loop (Supplementary Figure 2a, b). There were 149 MFS superfamily members in rice and several of them were predicted to transport various substrates (Supplementary Data 4). A phylogenetic tree was constructed for *OsCd1* along with all the MFS proteins in rice (Supplementary Fig. 3). It was clearly shown that the proteins facilitating the same substrate transport shared a closer phylogenetic relationship, while *OsCd1* clusters separately formed an unknown-function clade. Together with the yeast spot assay results, we deduced that *OsCd1* may form a new-function clade in MFS involving in Cd transport.

### *OsCd1* altered cadmium uptake and grain accumulation in rice

**Fig. 2:**
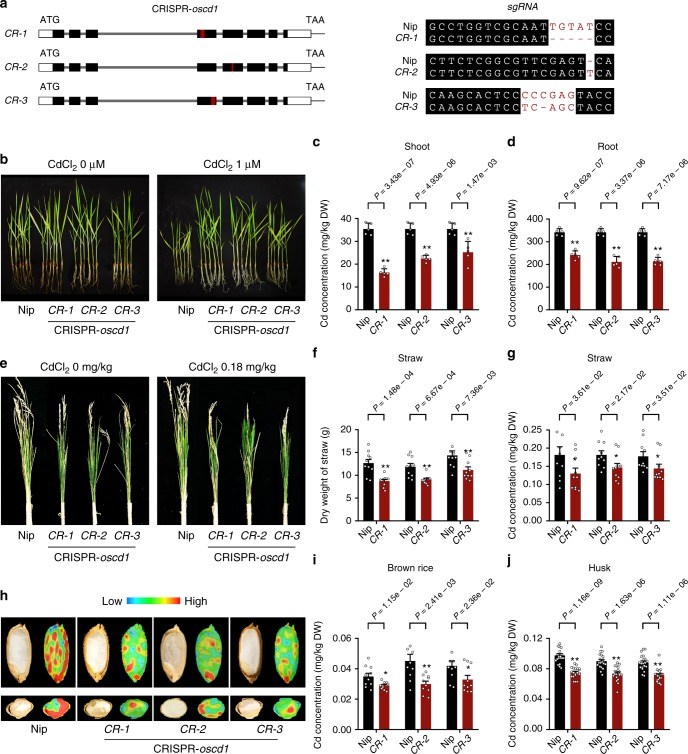
*OsCd1* contributes to the Cd uptake and grain accumulation in rice. **a** Left: Schematic diagram of three *CRISPR-oscd1* lines. Black rectangles represented exons of *OsCd1*, red rectangle represented the exon of target sequence. Right: Sequences of *CRISPR-oscd1* alleles *CR-1*, *CR-2*, and *CR-3*. sgRNA targets sequences were showed and deletions are indicated by red dashes. **b** Growth of wild-type rice and three *CRISPR-oscd1* lines after treating with and without 1 μM CdCl_2_ for 20 days. **c**, **d**, **g**, **i**, **j** Concentration of Cd in the shoot (**c**), root (**d**), straw (**g**), brown rice (**i**) and husk (**j**). **e** Growth of wild-type rice and three *CRISPR-oscd1* lines after treated with and without 0.18 mg kg^−1^ Cd in pot-test. **f** Dry weight of straw wild-type rice and three *CRISPR-oscd1* lines after treated with 0.18 mg kg^−1^ Cd in pot-test. **h** SR-*μ*XRF images of Cd distribution in the longitudinal (upper) and latitudinal (lower) sections of rice grain. The emission intensity of each pixel was normalized using the beam intensity as reference. The *CRISPR-oscd1* lines were shown in red and the wild-type line was shown in black. Error bars indicate standard deviation in the hydroponic experiment and standard error of mean in pot-test. Statistical comparison was performed by one-side *t*-test (*P < 0.05 and **P < 0.01). All data were compared with Nipponbare and designed with at least five replications and data points for all biological replicates are shown. Source data of Fig. 2c, d, f, g, i, and j are provided as a Source Data file

Considering the possible function of *OsCd1* in Cd transport, we transformed the *CRISPR/Cas9* constructs in rice callus and regenerated the transgenic plants to investigate the role of *OsCd1* in rice. Three Cas9-positive lines (*oscd1-cr1*, *oscd1-cr2,* and *oscd1*_*-*_*cr3*), which had either an insertion or deletion of one or few bases at the target sequences and disrupted the Cd transport ability of *OsCd1* (Supplementary Fig. 4), were selected for further research (Fig. 2a). In vegetative growth stage, the growth without CdCl_2_ treatment was not affected by the mutation of *OsCd1*. After being treated with 1 μM CdCl_2_ for 20 days, the *CRISPR-oscd1* lines displayed a better growth (Fig. 2b) and the Cd concentration both in root and shoot were much lower than that in the wild-type rice (Fig. 2c, d). We then grew them in the field until ripening to investigate the physiological role of *OsCd1* in grain Cd accumulation. At harvest, both the growth and the yield were affected by the disruption of *OsCd1* (Fig. 2e). The biomass of the straw (Fig. 2f) and the filled spikelet were reduced in the mutant lines due to decreased fertility (Supplementary Fig. 5). As to the Cd concentration, the result showed that there was a decrease Cd accumulation in the straw of three *CRISPR-oscd1* lines compared with the wild type (Fig. 2g). The synchrotron radiation microscopic X-ray fluorescence (SR-*μ*XRF) scanning was further used to reveal the spatial disparity in the distribution of Cd throughout the grain in situ. As it is shown in Fig. 2h, Cd enriched both in the endosperm and aleurone layer, and its concentration was in a lower level in *CRISPR-oscd1* lines than the wild type. Moreover, the Cd concentration was significantly decreased in brown rice and husk in three *CRISPR-oscd1* lines, which was in well agreement with the SR-*μ*XRF results above (Fig. 2i, j). The concentration of other micronutrients, including Zn, Mn, and Fe, were also detected in the *CRISPR-oscd1* lines. Compared with the wild-type rice, no difference in the concentration of Zn and Fe was observed but Mn, which is important in photosynthetic oxygen evolution in chloroplasts of plants, displayed a significant decrease in the *CRISPR-oscd1* lines (Supplementary Fig. 6).

**Fig. 3:**
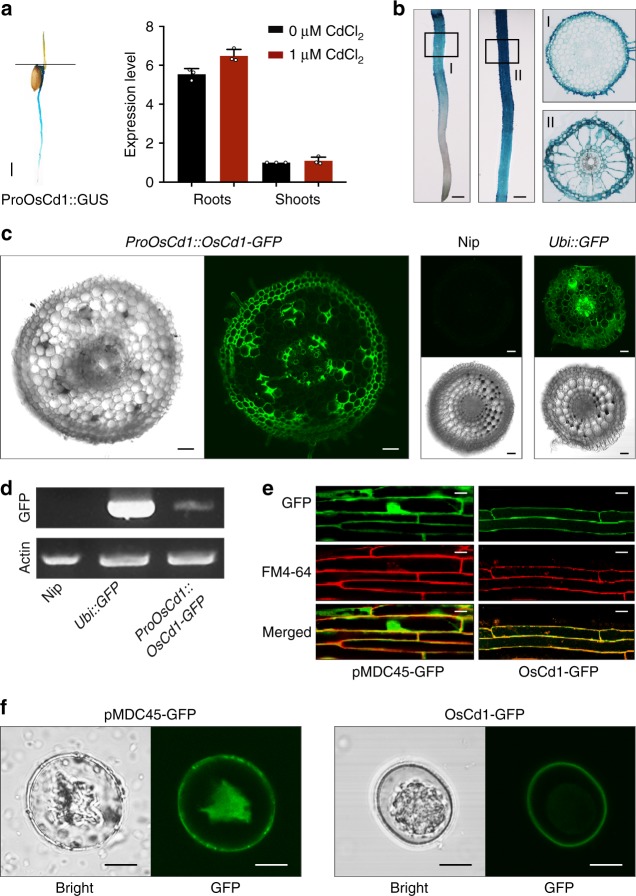
Expression pattern of *OsCd1* in rice. **a** Tissue-dependent expression of *OsCd1* at vegetative stage. Left: GUS histochemical assay of the transgenic plant with the GUS reporter driven by the *OsCd1* promoter. Right: the expression level of *OsCd1* in root and shoot with and without 1 μM CdCl_2_ treatment via qPCR. Bars = 2 mm. **b** Tissue-specific localization of *OsCd1* in the root of rice using the GUS reporter driven by the *OsCd1* promoter. Bars = 0.5 mm. **c** Tissue-specific localization of *OsCd1* in the root of rice using the GFP driven by the *OsCd1* promoter in cross frozen sections. Nip, negative control; Ubi::GFP, positive control. Bars = 10 μm. **d** The GFP transcriptional level shown by RT-PCR. Upper, *GFP*; Down, *Actin*. **e** Subcellular location of *OsCd1* in the root of rice observed by confocal laser scanning microscopy. The GFP fluorescence, fluorescence of FM4-64 and overlay of FM4-64 and *pMDC45*-GFP (left), *OsCd1*-GFP (right) were shown, respectively. Bars = 20 μm. **f** Subcellular location of *OsCd1* in root protoplasts. Bars = 10 μm. Source data of Fig. 3a and d are provided as a Source Data file

To determine the expression profile of *OsCd1* in rice, we analyzed the expression level from root and shoot at vegetative growth stage in rice via qPCR. The results showed that *OsCd1* was mainly detected in roots, which was consistent with the GUS histochemical assay, and not induced by CdCl_2_ (Fig. 3a). The cell-specific expression of *OsCd1* in rice root tissues was further analyzed using the GUS and GFP reporter fused to the *OsCd1* promoter. The promoter activity of *OsCd1* was mainly detected in the root exodermis, parenchyma in cortex, endodermis and stele cells (Fig. 3b–d and Supplementary Fig. 7). We then constructed the GFP fusion protein to examine the subcellular location of *OsCd1* in rice. The green fluorescence was mainly localized in the cytosol and nucleus of cells expressing GFP alone while the *OsCd1* fused with GFP was only observed at the periphery of the cells both in root cells and protoplast. The merged images of GFP and plasma membrane marker FM4-64 further confirmed the plasma membrane subcellular localization of *OsCd1* (Fig. 3e, f). Together with the *CRISPR-oscd1* results, it is suggested that the *OsCd1*, which was a plasma membrane protein in root, may be involved in Cd uptake and contributed to grain Cd accumulation in rice.

### SNP22 altered the cadmium transport ability of *OsCd1*

**Fig. 4:**
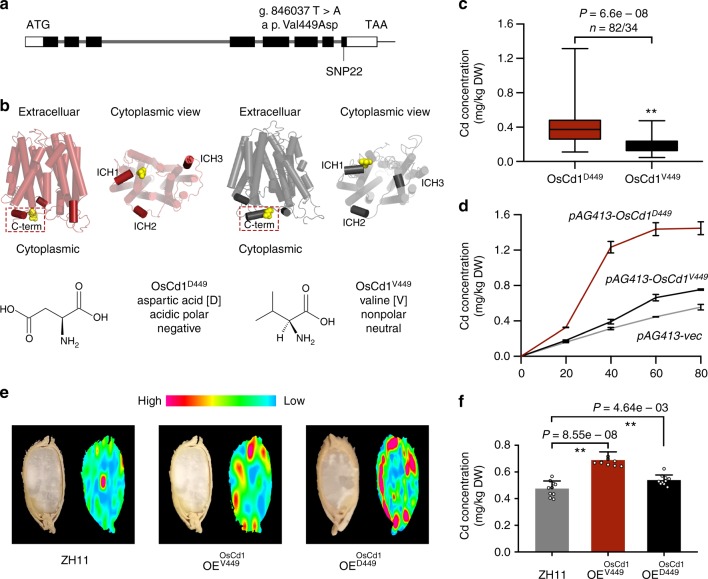
Functionally important amino acids in *OsCd1*. **a** Schematic diagram of gene structure and SNPs in *OsCd1*. **b** Predicted three-dimensional structural model of OsCd1. The structures of *OsCd1*^*D449*^ (red) were viewed parallel (left one) and perpendicular (right one) to the membrane, respectively, and so for *OsCd1*^*V449*^ (gray). Residue Asp449 in *OsCd1*^*D449*^ (red) and Val449 in *OsCd1*^*V449*^ (gray) were labeled at the cytoplasmic end respectively. ICH indicated intracellular helix. All structure figures were prepared with PyMol. **c** The grain Cd distribution of each genotype of *OsCd1* among rice natural variations group treated with 0.18 mg kg^−1^ in pot-test; *n* denoted the number of genotypes belonging to each group. In each box plot, the bold horizontal line indicates the median, the edges of the box represent the first and third quartiles, and whiskers extend to span a 1.5 interquartile range from the edges. **d** Cd accumulation in W303 expressing *OsCd1*^*D449*^ (red) and *OsCd1*^*V449*^ (black) yeast treated with 20, 40, 60, 80 μM CdCl_2_ for 24 h. Data from the transgenic lines were designed with three replications. **e** SR-*μ*XRF images of Cd distribution of ZH11, *OE-OsCd1*^*V449*^, and *OE-OsCd1*^*D449*^ lines in the rice grain. The emission intensity of each pixel was normalized using the beam intensity as reference. **f** Concentration of Cd in the brown rice of ZH11 (gray), *OE-OsCd1*^*V449*^ (black) and *OE-OsCd1*^*D449*^ (red) lines treated with 1.8 mg kg^−1^ in the field. Error bars indicate standard deviation. Statistical comparison was performed by one-side *t*-test (*P < 0.05 and **P < 0.01). Data from the transgenic lines were designed with ten replications and data points for all biological replicates are shown. Source data of Fig. 4c, d and f are provided as a Source Data file

The full-length sequences of 127 rice cultivars were used to investigate functional allelic variations in *OsCd1* locus. A total of 24 SNPs were identified in the *OsCd1* genomic region: 3 SNPs in the 5′UTR, 3 SNPs in the exon, 17 SNPs in the intron and 1 SNP in the 3′UTR, respectively (Supplementary Data 5). In specific, only the SNP22 (g. 846037 T>A), a missense mutation in eighth exon, resulted in a negative amino acid valine corresponding to a neutral one aspartic (a p. Val449Asp substitution) (Fig. 4a). *OsCd1* was a typical MFS protein with 12 transmembrane a-helices. Additionally, there were three extra intracellular helical domains (ICHs) on the intracellular side, one at the C terminus (ICH1) and the other two (ICH2 and ICH3) locating between the amino- and C-terminal TM bundles. The amino acid substitution V449D located on the C-terminal and around the ICH1 from the cytoplasmic face of the plasma membrane (Fig. 4b).

To find the haplotype between SNP22 with rice grain Cd accumulation, we then conducted the haplotype analysis for SNP22 and grain Cd accumulation in 127 rice cultivars. The results showed that the *OsCd1* can be classified into two genotypes based on the SNP22: *OsCd1*^*D449*^ with the A on SNP22 (*n* = 82) and *OsCd1*^*V449*^ with the T on SNP22 (*n* = 34). Statistically, lines with *OsCd1*^*D449*^ have an ~2-folds grain Cd concentration compared to those with *OsCd1*^*V449*^ (*P* = 6.68 × 10^−8^) (Fig. 4c) but with no obvious difference in expression levels and subcellular location (Supplementary Fig. 8 and 9). We further analyzed the Cd concentration in *OsCd1*^*V449*^ and *OsCd1*^*D449*^ transgenic yeast strains to investigate if the variation caused by SNP22 might alter the Cd transport ability of *OsCd1*. As shown in Fig. 4d and Supplementary Fig. 10, *OsCd1*^*D449*^ resulted in a higher Cd concentration compared to *OsCd1*^*V449*^ under Cd treatment. Meanwhile, the expression level showed that there was no significant difference between the *OsCd1*^*V449*^ and *OsCd1*^*D449*^ transgenic lines with or without Cd treatment (Supplementary Fig. 11). To determine whether SNP22 led to alteration of grain Cd accumulation in rice, we generated transgenic lines by overexpressing the *OsCd1*^*V449*^ and *OsCd1*^*D449*^ in a ZH11 background. Both the *OE-OsCd1*^*V449*^ and *OE-OsCd1*^*D449*^ transgenic lines accumulated more Cd compared to ZH11. In addition, the transgenic lines of *OE-OsCd1*^*V449*^ showed a significantly less Cd accumulation ability in rice grain than *OE-OsCd1*^*D449*^ (Fig. 4e, f). We then conducted the functional complementation assay by crossing both NILs with the *OsCd1*^*D449*^ and *OsCd1*^*V449*^ to the *CRISPR-oscd1* knockout lines and treated the F1s with 1.8 mg kg^−1^ Cd in the field. At harvest, we measured the Cd concentration in the grain and it was observed that a higher level in F1s with *OsCd1*^*D449*^ than that of F1s with *OsCd1*^*V449*^ (Supplementary Figure 12). Together with the results in yeast systems, we concluded that the SNP22, resulting in a change of encoded amino acid, altered the Cd transport ability of *OsCd1*.

### *OsCd1*^*V449*^ introgression reduced grain cadmium accumulation

**Fig. 5:**
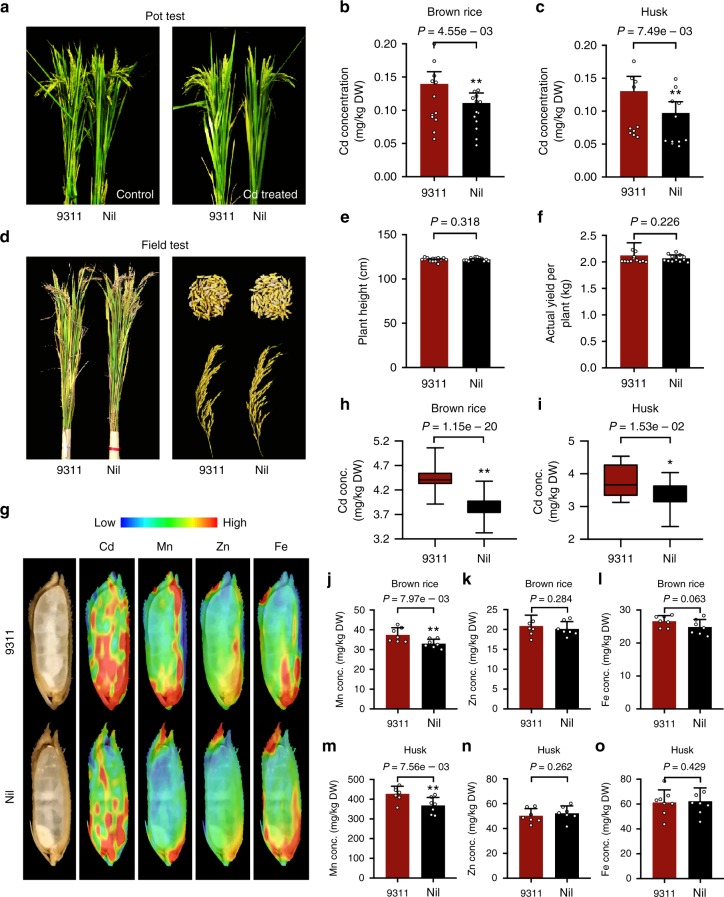
*OsCd1*^*V449*^ introgression reduces grain Cd accumulation. **a** Growth of 9311 and NIL after treated with or without 0.18 mg kg^−1^ Cd in pot-test. **b**, **c** Concentration of Cd in the brown rice and husk. Error bars indicate standard error of mean; statistical analysis was inspected using one-side paired *t*-test (*P < 0.05 and **P < 0.01). **d** Growth and grain morphologies of 9311 and NIL after treated with 1.8 mg kg^−1^ Cd in the field. **e** Plant height of 9311 and NIL. **f** Actual yield per plot of 9311 and NIL. **g** SR-*μ*XRF images of Cd, Mn, Zn, Fe distribution in the longitudinal sections of rice grain. The emission intensity of each pixel was normalized using the beam intensity as reference. **h**–**o** Concentrations of Cd, Mn, Zn, Fe in the brown rice (**h**, **j**–**l**) and husk (**i**, **m**–**o**). 9311 were shown in red and the NIL was shown in black. In each box plot, the bold horizontal line indicates the median, the edges of the box represent the first and third quartiles, and whiskers extend to span a 1.5 interquartile range from the edges. Error bars indicate standard deviation. Statistical analysis was inspected using one-side *t*-test (*P < 0.05 and **P < 0.01). All data were compared with 9311 and designed with at least five replications and data points for all biological replicates are shown. Source data of Fig. 5b, c, e, f, and h–o are provided as a Source Data file

Considering that *OsCd1*^*V449*^ exhibits lower Cd transport ability than that of *OsCd1*^*D449*^, we generated near-isogenic line (NIL) by the introgression *OsCd1* ^*V449*^ of Nipponbare (*japonica*) into the background of 9311 (*indica* with *OsCd1*^*D449*^ genotype) for further research (Supplementary Figs. 13a and b). The effect of the allele makeup at *OsCd1* on grain Cd accumulation was firstly investigated in 0.18 mg kg^−1^ treated pot-grown rice at greenhouse. As it is shown in Supplementary Fig. 13c and d, 9311 displayed higher Cd concentration in rice grain than the female parent Nipponbare lines. With the introgression of *OsCd1*^*V449*^, the Cd accumulation of NIL was significantly reduced both in brown rice and husk compared with the 9311 backgrounds with no apparent difference in growth (Fig. 5a–c).

To evaluate the effect of *OsCd1*^*V449*^ allele in vitro, we selected the Cd-polluted fields at Changsha (Hunan Province, China, E112°, N28°)^18^ with 1.8 mg kg^−1^ Cd to perform the field trial. According to the newly published National Standard of the People’s Republic of China: Soil environment quality Risk control standard of soil contamination of agricultural land (GB15618-2018), the risk intervention values for Cd is set to 1.5 mg kg^−1^ (pH ≤ 5.5) or 2.0 mg kg^−1^ (5.5 < pH ≤ 6.5)^34^. The experimental level of Cd used in this filed is representative of a general situation of Cd-contaminated paddy fields in China. At harvest, there was no apparent difference in plant growth between the NIL and 9311. Moreover, the filled spikelet and actual yield per plot were also not affected by the introgression of *OsCd1*^*V449*^ (Fig. 5d–f). Notably, the grain Cd concentration was detected to be reduced in NIL (Fig. 5h, i). According to the SR-*μ*XRF results, the Cd distribution in whole grain of NIL, especially the edible part endosperm, were significantly less than that in 9311 (Fig. 5g). For other micronutrients, except for a slightly decrease of Mn, no obvious change in the concentration of Zn and Fe was observed between 9311 and NIL (Fig. 5g, j–o). Additionally, when *OsCd1*^*V449*^ was introduced into GUICHAO-2, another *indica* cultivar widely cultivated in south China, the grain Cd accumulation was also substantially decreased (Supplementary Fig. 14). These results demonstrated that the allelic effect of *OsCd1*^*V449*^ contributed to the reduction of Cd in rice and, furthermore, may have a widely potential application value in rice genetic improvement.

### *OsCd1* diverged between *indica* and *japonica* subspecies

**Fig. 6:**
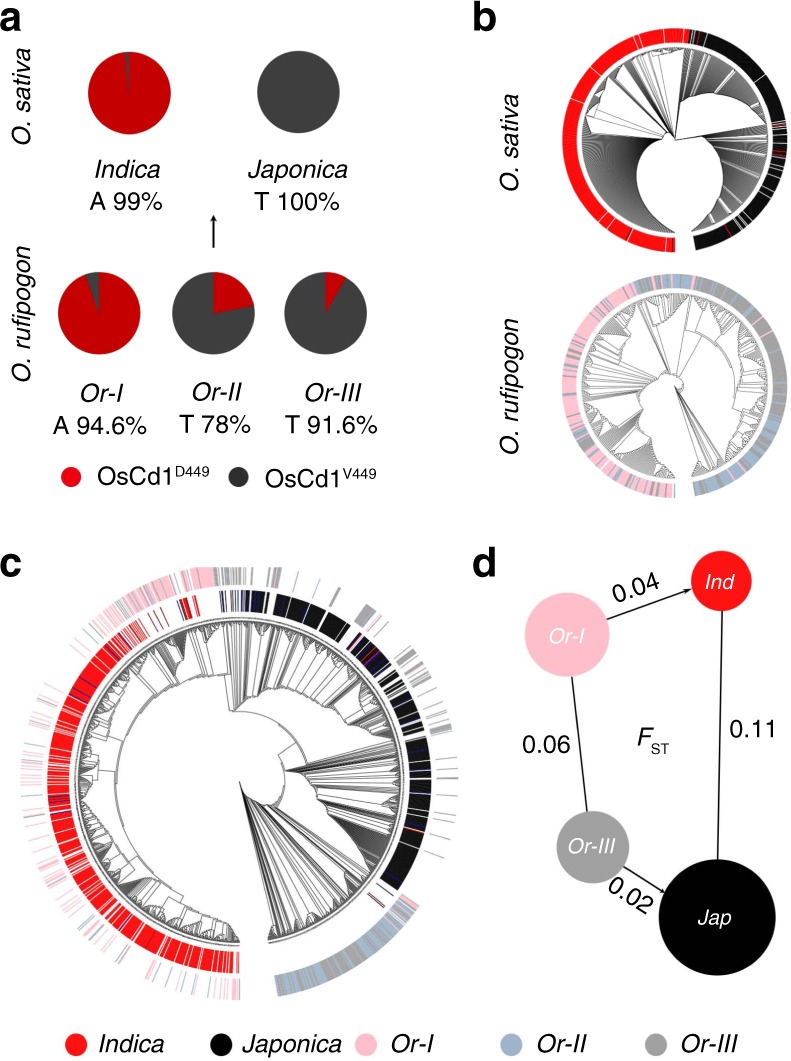
Genotypes and phylogenetic analysis of *OsCd1*. **a** The spectrum of allele frequencies at the causal polymorphisms of *OsCd1* in *O. rufipogon* (Or-I, Or-II and Or-III) and *O. sativa* (*japonica* and *indica*). **b** Phylogenetic tree of the full population calculated from the SNPs of *OsCd1* in *O. sativa* (*indica* and *japonica* subspecies, up) and *O. rufipogon* (Or-I, Or-II and Or-III, down). **c** Phylogenetic tree of the full population calculated from the SNPs of *OsCd1* in *O. rufipogon* (outer ring) and *O. sativa* (inner ring). **d** Illustration of genetic diversity and population differentiation in *O. rufipogon* and *O. sativa*. The *F*_st_ values between the groups were indicated. ind, *indica*; jap, *japonica*. Or-I, Or-II and Or-III are colored in pink, light blue and gray respectively; *indica* and *japonica* subspecies are in red and black, respectively

Interestingly, it was also observed that the *OsCd1* in most of the *indica* accessions was with *OsCd1*^*D449*^ (A at SNP22 site) and that in most of the *japonica* accessions was with the *OsCd1*^*V449*^ (T at SNP22 site) (Supplementary Table 2). In special, the grain Cd accumulation level was significantly lower in the *indica* cultivars with *OsCd1*^*V449*^ than that of *OsCd1*^*D449*^ (Supplementary Fig. 15), indicating that the Cd accumulation of *indica* cultivars with *OsCd1*^*V449*^ also fitted the regular pattern for *japonica* rice. To explore the phylogenetic relationship of this allele, we then used whole-genome sequencing data for a large panel of accessions^35^ containing 446 *O. rufipogon* accessions and 950 *O. sativa* varieties to determine the ancestral states of the SNP22 in *OsCd1*. Phylogenetic analysis using the 950 cultivar rice accessions showed that all the *japonica* accessions have nucleotide T (genotype *OsCd1*^*V449*^) at SNP22 while 99% of the *indica* accessions have nucleotide A (genotype *OsCd1*^*D449*^). Moreover, both the nucleotide A and T in SNP22 retained in its ancestor *O. rufipogon* (Fig. 6a, Supplementary Table 3).

Based on phylogenetic tree and PCA analysis, it was demonstrated that *OsCd1* diverged between *indica* and *japonica* subspecies in cultivated rice and between subgroup Or-I and Or-III in wild rice (Fig. 6b). Further analysis revealed that *OsCd1* in *indica* was descended from *O. rufipogon* Or-I, which mainly distributes in South and Southeast Asia, while that in *japonica* was from Or-III, which mainly distributes in China^19^ (Fig. 6c and Supplementary Figure 16). The level of population differentiation (*F*_st_) was estimated to be 0.06 in *O. rufipogon* and increased to 0.11 in *O. sativa* during domestication (Fig. 6d). No significant Tajima’s *D* values were observed for *japonica* (Tajima’s *D*-1.16) and *indica* (Tajima’s *D*-1.32) cultivars, indicating that the divergence of *OsCd1* between *indica* and *japonica* did not subject to selection and derived from the divergence of their progenitor gene pools hundreds of thousands of years ago.

## Discussion

Reducing rice grain Cd accumulation is an important task for decreasing risks to human health. Rice absorbed Cd from soil by roots and finally transferred then into grain via transport systems. Thus, identifying the genetic components underlying Cd transport is of great importance to reduce Cd accumulation in rice grain. More recently, GWAS has been successfully used for dissecting loci in several important agronomic traits^28–32^ and was a promising strategy for finding genetic variants underlying the grain Cd accumulation in rice.

In this study, we analyzed the rice grain Cd contention in 127 varieties and performed GWAS to dissect the valuable genetic components underlying rice grain Cd accumulation (Fig. 1a–d). As a result, QTL3, which showed the greatest contribution to the phenotypic variation, was selected for further research. Totally, 126 predicted genes with the annotation, such as transporter and transcription factor, were identified and may be the possible functional components in QTL3 (Supplementary Data 2). Using a composite bioinformatics tool-box method, *OsCd1*, which belongs to the major facilitator superfamily (MFS), was detected to be associated with rice grain Cd accumulation on the whole-genome scale and confirmed to mediate Cd influx into cell in yeast system (Fig. 1e, f).

MFS is the largest group of secondary active membrane transporters, and its members transport a diverse range of substrates^36^. Recently, MFS proteins were reported to be functioned as transporters of some metal elements such as *Arabidopsis* zinc (Zn) transporter ZIF1^37^ and *Arabidopsis* cesium (Cs) and potassium (K) transporter ZIFL2^38^, whereas the role of MFS proteins in Cd transport was still not clear. Here, we characterized the MFS protein OsCd1 as a possible Cd transporter gene in rice and the disruption of *OsCd1* resulted in a decrease of Cd accumulation in root, shoot, and grain (Fig. 2). Furthermore, subcellular and tissue localization showed that *OsCd1* was a plasma membrane protein and mainly expressed in the root exodermis, cortex, endodermis, and stele cells tissue (Fig. 3). We could then speculate that *OsCd1* may mediate the Cd uptake in rice root and, ultimately, contribute to grain Cd accumulation. Together with the results in transgenic yeast and *CRISPR-oscd1* lines, we thus deduced that *OsCd1* may form a new-function clade in MFS involving in Cd transport. The exact role of *OsCd1* in Cd accumulation, however, needed to be further explored.

The identification of *OsCd1* enables us to reduce Cd accumulation in rice grain by transgenic techniques. However, the disruption of *OsCd1* is likely to result in a decrease of growth at harvest (Fig. 2e and Supplementary Fig. 5). As it was reported previously, *OsCd1* (*ASL*) shares 93.2% similarity to the gene *ASY*, which regulates early shoot development in rice. The absent of *ASY* showed various shoot abnormalities at the early vegetative stage. *OsCd1* (*ASL*) exists in the rice genome and is supposed to have redundant functions. For the reason that the *OsCd1* (*ASL*) expression level increased at later stages, it was speculated that *OsCd1* (*ASL*) may be involved in the shoot development of later stages^39^. In addition, it was also observed that *OsCd1* influences the Mn accumulation while the exact mechanism needs to be further investigated (Supplementary Fig. 6). Mn is a required trace mineral for all known living organisms and functions as cofactors for a large variety of enzymes with many functions^40,41^ and the disruption of *OsCd1* was likely to influence the rice growth at harvest.

Considering the fact that the disruption of *OsCd1* has a negative impact on plant growth and reduces yield at harvest, it is thus not an optimal strategy to establish low-Cd rice by simply knocking out *OsCd1*, while selection of superior alleles of *OsCd1* may be a more effective strategy for genetic improvement of rice. We then conducted research to excavate natural variation allele in *OsCd1*. Based on the sequences, we identified a nonsynonymous mutation (SNP22), locating at the ICH1 of C-terminal (Fig. 4a, b), could cause a charged amino acid D449 substituting to an uncharged one V449. Typically, MFS protein possesses 12 transmembrane a-helices with both their N- and C-terminal located in the cytoplasm^42–46^. During the transport reaction cycle, some amino acids on N- and C-terminal form hydrogen bonds with the substrate and translocate the substrate via the “rocker-switch” mechanism^42,47,48^. N- and C-terminal, especially the charged or polar residues in the ICH domain, are likely to have a critical role to close the transporter on the intracellular side during the alternating access cycle^49^. When the V449 was substituted to D449 in SNP22, the polarity change caused a relatively stronger Cd transport ability in *OsCd1*^*D449*^ compared with *OsCd1*^*V449*^ (Fig. 4d). The yeast essay, together with the complementary experiments with over-expression promoter indicated that the Cd transport ability varied in two alleles (Fig. 4e, f). Considering native promoter, which is a better strategy for functional complementation, was not used, we thus crossed the alternative alleles to the *CRISPR-oscd1* lines and the Cd transport ability variation was furtherly confirmed (Supplementary Fig. 12). The difference in Cd transport ability, finally, endowed the *OsCd1*^*D449*^ rice cultivars with a higher grain Cd accumulation ability than the cultivars with *OsCd1*^*V449*^ (Fig. 4c).

In addition, no tissue expression level and subcellular location differences were observed between the *OsCd1*^*V449*^ and *OsCd1*^*D449*^ (Supplementary Figs. 8 and 9). We thus regarded SNP22 in *OsCd1* as a critical allele contributing to the divergence in rice grain Cd accumulation and tried to apply the functional allele *OsCd1*^*V449*^ for marker-assisted breeding.

Two rice cultivars, 9311 and GUICHAO-2, which showed a relatively higher grain Cd content compared with Nipponbare and Guihuahuang, were selected as the background of NIL. When the *OsCd1*^*V449*^ allele was introduced into them, lower grain Cd accumulation NILs were obtained (Fig. 5b, c, h, i and Supplementary Fig. 14). Meanwhile, it is noteworthy that, different from *CRISPR-oscd1* lines, which resulted in a complete loss of gene function, the NIL with lower grain Cd accumulation has no apparent difference in plant growth and spikelet fertility compared with 9311, though the Mn accumulation in NIL was slightly decreased (Fig. 5d–f and Supplementary Fig. 14). This suggests *OsCd1*^*V449*^ a great potential application in low-Cd rice cultivation.

Phylogenetic and population genetic analyses demonstrated the natural variation in *OsCd1* diverged between *indica* and *japonica* subspecies: the *japonica* accessions with genotype *OsCd1*^*V449*^ displayed a relatively lower Cd accumulation in rice grain than the *indica* accessions with genotype *OsCd1*^*D449*^ (Fig. 6). This was different with *OsNramp5*, a major Cd transporter in rice. In *OsNramp5*, the frequency of the two missense variation SNPs in *OsNramp5* showed almost no difference in *indica* and *japonica*, indicating it contributed little to the Cd divergency between *indica* and *japonica* rice (Supplementary Fig. 17). In Asia, the cultivated rice is mainly classified into *indica* and *japonica* subspecies. *Indica* cultivars, which are the major food and widely grown in the South and Southeast Asia, generally exhibit higher grain Cd accumulation than *japonica* cultivars^50,51^ (Supplementary Fig. 1c). Since Cd is one of the most serious soil heavy metal pollutant in these regions^52,53^, the ingestion of *indica* rice grain brings an unneglectable risk of Cd to people living there. In the previous study, three *osnramp5-1* rice mutants were developed by carbon ion-beam irradiation strategy for marker-assisted selection of low-Cd cultivars^11^. However, the parent of these three rice mutants is Koshihikari, which is the main cultivar in Japan but not in China and other Southeast Asian countries. *OsCd1*^*V449*^ exists in most of the *japonica* cultivars which makes it a wider parent selection range in low-Cd cultivar breeding. By integrating the low-Cd allele *OsCd1*^*V449*^ into *indica* cultivars, the grain Cd accumulation had been successfully reduced on the Cd-contaminated paddy fields in Hunan province, where is considered as a typical Cd-contaminated area in China and has suffered from Cd contamination for many years. This finding, along with the function characterizing of natural variation of *OsCd1*, emphasizes the potential application value of *OsCd1*^*V449*^ in low-Cd rice cultivation, especially in *indica* cultivars.

## Methods

### GWAS analysis and gene annotation

A diverse worldwide collection of 127 *O. sativa* accessions including both landraces and elite varieties was obtained from a core/mini-core collection of *Oryza sativa* L. for GWAS analysis^33^. Each accession was germinated and planted in cultivation pot treated with 1.8 mg kg^−1^ Cd (Beijing, China; 39.9°N, 116.3°E). The grain was collected after harvest to test the Cd concentration of each genotype. In total, we used 3,291,150 SNPs with a minor allele frequency of >0.05 to carry out GWAS. Association analysis using a mix model was performed with the GAPIT software package. The top three principal components were used as fixed effects, and the matrix of genetic similarity based on simple SNP matching coefficients was used to model the variance-covariance matrix of the random effect. The analyses were performed in GAPIT and the parameters for each trait were optimized automatically.

Permutation tests were used to help define the genome-wide significant *P*-value threshold^54^. For all the examined traits, we reshuffled the original phenotype data, and then performed association analysis using GAPIT with the same parameters. There ought to be no real associations between the SNPs and the simulated phenotypes, so all the SNPs passing the permutation threshold should be positives. After conducting 1000 permutation analyses, we clearly found that the permutation threshold varied across genomic region and some of the association signals even passing the significant cutoff 10^−5^. Thus, we adopted a threshold *P* = 10^−5^ at genome-wide level.

For QTL and gene annotation, we firstly selected the SNP signal that passing the threshold at *P* = 10^−5^, to check if the distance to the adjacent SNP was less than 170 kb^32^. We then merged it into the same QTL, all the genes locating in the QTL region were predicted by the Rice Genome Annotation Project (MSU-RGAP, Nipponbare version 6.1, http://rice.plantbiology.msu.edu/cgi-bin/gbrowse/rice/) and annotated by gene ontology (GO) Slim annotation with the keyword membrane and transport.

### Primers

The sequences for all primers used in this study are listed in Supplementary Table 4.

### Cadmium and manganese transport activities assay in yeast

RNA was extracted from *CRISPR-oscd1*, 9311 and Nipponbare accessions. For candidate gene selection assay, the coding sequence of *C1*, *C2*, *C3*, and *C4* was amplified from 9311 cDNA. The haplotypes *OsCd1*^*V449*^ and *OsCd1*^*D449*^ were amplified from 9311 and Nipponbare cDNA, respectively. The *oscd1-CR1*, *oscd1-CR2* and *oscd1-CR3* were amplified from *CRISPR-oscd1*, respectively. The sequences were then cloned into *pAG413GAL* vector to construct yeast expression vector. The constructed vectors along with the empty vector *pAG413GAL* were transformed into the wild-type *Saccharomyces cerevisiae* W303 (*MATa his3-1 met15-0 trp1-1 ura3*, Laboratory preservation), *Δycf1* (MATa *ura3 leu2 his3-1 met15-0 YDR135c::kanMX4*, Laboratory preservation) and *Δsmf1* (MATa *his3-1 leu2 met15-0 ura3 YOL122c::kanMX4*, Chen Caiyan’s Laboratory preservation).

Polyethylene glycol (PEG)/LiAc-based method was used for preparing and transforming competent yeast cells^55^. Yeast cells were grown at 30 °C in YPAD medium and then treated with 100 mM LiAc to make yeast competent cells. The mixture of ~200 ng μl^−1^ plasmid, 240 μl PEG solutions, 26 μl 1 M LiAc and 10 μl Carrier DNA were added into the yeast competent cells and incubated at 30 °C for 30 min. Then the mixture was heat shocked at 42 °C for 20 min. Yeast transformants were cultured in synthetic defined (SD) medium without histidine at 30 °C for 48 h.

For plate growth tests, yeast transformants were diluted to an OD_600_ of 1.0, 0.1, 0.01, and 0.001 step by step with sterile water. Then the yeast transformants were spotted on SD-His plates (with 2% (w v^−1^) galactose/glucose) with or without 30 μM CdCl_2_ or 5 mM EGTA, respectively. The yeast carrying empty vector was used as control. Plates were incubated for 2–3 d at 30 °C for phenotype observation. All the assays were performed at least three times.

For the Cd and Mn concentration determination, the yeast transformants were cultured overnight with liquid SD medium (with 2% galactose) to OD_600_ = 1 with 200 rpm at 30 °C. Then the yeast transformants were treated with 20, 30, 40, 60, 80 μM CdCl_2_ for 24 h or 5 mM EGTA for 48 h, respectively. The yeast strain carrying empty vector was used as control. All the yeast samples were collected and washed with sterile water for three times. Then all the samples were dried at 80 °C in an oven for further Cd and Mn concentration determination. All the assays were performed at least three times.

### 3D structure modeling and protein structure analysis

The *OsCd1* sequence was analyzed by Phyre2^56^ and secondary structure was displayed using TMRPres2D^57^ to determine the position of the transmembrane helices. Three-dimensional model of *OsCd1*^*V449*^ and *OsCd1*^*D449*^ were constructed using the Phyre2 and MODELLER software^58^. Three high-resolution protein structures GlpT (PDB entry 1pw4), YajR (PDB entry 3wdo), and POT (PDB entry 4iky) were used as templates simultaneously in the comparative modeling procedure. Three-dimensional models were visualized within PyMOL^59^.

### Phylogenetic reconstruction of *OsCd1*

In total 149 protein sequence of MFS members from rice were obtained from Transport Database (http://www.membranetransport.org/transportDB2/index.html). Multiple-sequence alignment was optimized by MUSCLE. The phylogeny of *OsCd1* in the MFS was constructed by MEGA7^60^ using the neighbor-joining method with No. of differences model, pairwise deletion for missing data and 1000 bootstrap pseudoreplicates. The software EvolView^61^ was used for visualizing the phylogenetic trees.

### *CRISPR-oscd1* mutant lines and function complementary essay

The sgRNA-Cas9 and *pUN1301-OsCd1* plant expression vectors were constructed^62^. The oligos used in constructing the sgRNA vectors for *OsCd1* are listed in Supplementary Table 5. The rice variety Nipponbare (*Oryza sativa* L. ssp. *japonica*) was used as the hosts in agrobacterium-mediated transformation for *CRISPR-oscd1* mutant and ZH11 (*Oryza sativa* L. ssp. *japonica*) was used as the hosts in agrobacterium-mediated transformation for *OE-OsCd1* lines (Hangzhou Biogle Co., LTD). The transgenic rice lines were grown in the paddy field in Hainan (18.48° N, 110.02° E, China), during normal rice-growing seasons. For *CRISPR-oscd1* mutant lines, mature seeds were collected from T_0_ plants and geminated for genotyping. Total DNA was isolated from transgenic plants and PCR was performed to amplify the genomic region surrounding the CRISPR target sites using the specific primers. The PCR fragments were directly sequenced by Sanger method to identify mutations. The *CRISPR-oscd1* lines were then used for crossing with NIL and 9311 respectively and the T_1_ plants was the functional complementary experiments. For the *OE-OsCd1* lines, the GUS marker was used for the selection of pure rice lines in T_2_ plants and then the pure rice lines were used for further experiments.

### Hydroponic experiments

To conduct the hydroponic experiments in vegetative stage, rice seeds of wild-type rice and *CRISPR-oscd1* lines were sown in a germination box with water in the dark. Then the 3-days rice were transferred to Kimura B solution containing (NH_4_)_2_SO_4_, MgSO_4_·7H_2_O, KNO_3_, Ca(NO_3_)_2_·4H_2_O, and KH_2_PO_4_ and the micronutrients MnCl_2_·4H_2_O, H_3_BO_3_, (NH_4_)_6_Mo_7_O_24_·4H_2_O, ZnSO_4_·7H_2_O, CuSO_4_·5H_2_O, and Fe-EDTA, pH 5.6. After growing in Kimura B solution 5 days, 1 μM CdCl_2_ were added and the seedings were treated with CdCl_2_ for 20 days. The plants were cultivated in the growth chamber at a temperature of 30 °C and the nutrient solution was replaced every 2 d. Then the shoot and root were collected separately with five replications to determine the Cd concentration. All the assays were performed at least three times.

### Pot and field experiments

Pot experiments were carried out at the greenhouse of Institute of Botany, Beijing. Three-week seedlings of Nipponbare, *CRISPR-oscd1* lines, 9311 and NILs pre-cultured hydroponically were transplanted to the pot with 0.18 mg kg^−1^ Cd in May and harvested at September. All experiments were repeated with 30 biological replicates.

Field tests of 9311 and the NIL were performed during May to September in 2017 and 2018. All the cultivars were planted in the Cd-polluted paddy fields at Institute of Subtropical Agriculture, CAS. The soil Cd concentration was 1.80 mg kg^−1^, pH 5.4. Randomized complete block design was applied in the field experiments. Each sample was assigned with three replicates. All the seedlings were transplanted at a spacing of 17 cm within rows and the distance between two rows was 20 cm. Following method was used to measure the agronomic traits including plant height, filled spikelet rate and grain yield per plant after harvest. Plant height was the length of main tiller; Filled spikelet rate was calculated as follows: filled grains/(unfilled grains + filled grains) × 100%; The grain yield was determined by weighing all the grains of one plant after drying at 50 °C in a lab oven for 8 h. The grain metal concentration was determined as described follows.

### Determination of metal accumulation using ICP-MS

The content of total Cd, Zn, Mn, and Fe in the yeast and plant samples were determined by Inductive Coupled Plasma Emission Spectrometer (ICP-OES) (iCAP6300, Thermo Electron Corp., MA, USA). All samples were dried at 80 °C for 6 h. Dried samples were digested using 2 ml of concentrated HNO_3_ overnight. Sample digestion was carried out by heating block at 200 °C for 8 h. After cooling, the digested solution was diluted to 15 ml with deionized water and filtered through 0.22 μm cellulose acetate membranes filters. All digestions were performed in triplicate. The blank HNO_3_ was used as the negative control and the certified standard material sample (CRM rice; GBW100348) was used as the positive control. All the assays were performed at least three times.

### In situ analyses using synchrotron X-ray fluorescence

Metal distribution in brown rice was evaluated using micro-X-ray fluorescence (SR-*μXRF*) method, which performed at 4W1B beamline, Beijing synchrotron Radiation Facility. The longitudinal and latitudinal sections of rice samples were prepared for SR-*μXRF* analysis. The incident X-ray energy is 15 keV and the spot size diameter is 50 μm. Si (Li) solid state detector was used to detect X-ray fluorescence emission lines. Step-mode with 100*100 μm was used to acquire the two-dimensional mapping of rice grain samples and each step’s live time is 30 s. After sample mapping, the data processing was performed using PyMca package^63^ and Origin 8.

### Quantitative real-time PCR

Nipponbare varieties, 20 *japonica* and 20 *indica* cultivars were treated with and without 1 μM CdCl_2_ for 24 h, respectively. Total RNA of the root, shoot and the whole plant was extracted using TRIzol reagent (Invitrogen) and then treated with DNase I. After DNaseI digest, ~2 μg RNA was converted to cDNA and the product was used as templates for the qPCR. Water and the cDNA without DNaseI digestion were used as negative control. qPCR was performed on an ABI 7500 instrument using SYBR Green PCR Master mix following the manufacturer's instructions (Takara, RR420). *Oshiston* gene were used as internal standards, and normalized relative expression was calculated by the ΔΔCt (cycle threshold) method. The primers used in qPCR are listed in Supplementary Table 4.

### Tissue expression assay

For histochemical analysis of GUS activity, the upstreaming 2.5 kb genomic fragment of *OsCd1* was regarded as the *OsCd1* promoter and cloned into pCAMBIA1391 to generate OsCd1_promoter_::GUS vector. The vectors were then transformed into Nipponbare and the transgenetic rice were sampled to detect GUS expression. To determine the activity of GUS, the samples were incubated into the solution containing 1 mM X*-*gluc, 10% methanol, 0.5% Triton X-100 and 50 mM Na_3_PO_4_ at 37 °C for 24 h. After being stained, 75% ethanol was applied to remove chlorophyll. Resin semi-thin sections were prepared for image collection to determine GUS activity.

For the GFP observation, the *OsCd1*’s promoter was cloned into pMDC45 and then the OsCd1_promoter_::GFP vector was then transformed into Nipponbare. The transgenetic rice root tissues were prepared into the frozen sections and then the confocal laser scanning microscope (FluoView FV1000, Olympus) was used to observe the location of *OsCd1*. Nipponbare was used as the negative control and Ubi::GFP transgenetic rice was used as the positive control.

### Subcellular-localization assay

The CDs of *OsCd1*^*V449*^ and *OsCd1*^*D449 *^were fused in frame with GFP via cloning into the binary vecor pMDC45. The resulting vectors pMDC45-*OsCd1*^*V449*^ were transformed into Nipponbare (*Oryza sativa* L. ssp. *japonica*) and used as hosts in agrobacterium-mediated transformation method. For FM4-64 staining, rice root was transferred to 2.5 μM FM4-64 diluted in 1/2 MS medium for 3 min. Besides, the protoplasts were isolated from root and the pMDC45-*OsCd1*^*V449*^ and pMDC45-*OsCd1*^*D449*^ were transiently expressed in the root protoplasts for GFP observation. The subcellular localization and co-localization were evaluated using a confocal laser scanning microscope (FluoView FV1000, Olympus).

### Population genetics analysis

12 kb of the sequence centered on *OsCd1* in 446 *O. rufipogon* accessions and 950 *O. sativa* varieties were obtained from rice HapMap3 data set (http://server.ncgr.ac.cn/RiceHap3/). SNPs in the 12-kb region were used for the *OsCd1* variety phylogenetic reconstruction analysis by MEGA7 using the neighbor-joining method with No. of differences model, pairwise deletion for missing data and 1000 bootstrap pseudoreplicates. Principal component analysis of the SNPs was performed using the software TASSEL. The level of population differentiation (*F*_st_)^64^ and Tajima’s D^65^. Statistic was calculated by a custom PERL script using a 1000 bp window. The *F*_st_ between Or-I and Or-III in *O. rufipogon* and between *japonica* and *indica* of *O. sativa* was calculated, respectively.

### NIL construction

*Oryza sativa* near-isogenic line (NIL) were selected from a BC_5_F_2_, which was generated by crossing between *japonica* variety Nipponbare × *indica* variety 9311 then backcrossing to 9311, respectively. 120 SSR markers distributed evenly throughout 12 chromosomes were used for identification and selection of the candidate lines containing the target donor segment. The size of the introgression fragment in the NIL was about 1 Mb between RM3413 and RM3372.

### Reporting summary

Further information on research design is available in the Nature Research Reporting Summary linked to this article.

## Supplementary information

Supplementary Information

Reporting Summary

Description of Additional Supplementary Files

Supplementary Data 1

Supplementary Data 2

Supplementary Data 3

Supplementary Data 4

Supplementary Data 5

Source Data

## Data Availability

Data supporting the findings of this work are available within the paper and its Supplementary Information files. A reporting summary for this Article is available as a Supplementary Information file. The datasets generated and analyzed during the current study are available from the corresponding author upon request. The source data underlying Figs. 1f, 2c, d, f, g, i, j, 3a, d, 4c, d, f, 5b, c, e, f, and h–o, and Supplementary Figs. 1c, 4b, 5b, 6a–c, 9, 10b, c, 11, 12b, 13c, d, 14b, and 15 are provided as a Source Data file.
