# Author Correction: Variation of a major facilitator superfamily gene contributes to differential cadmium accumulation between rice subspecies

**DOI:** 10.1038/s41467-019-10977-5

**Published:** 2019-07-19

**Authors:** Huili Yan, Wenxiu Xu, Jianyin Xie, Yiwei Gao, Lulu Wu, Liang Sun, Lu Feng, Xu Chen, Tian Zhang, Changhua Dai, Ting Li, Xiuni Lin, Zhanying Zhang, Xueqiang Wang, Fengmei Li, Xiaoyang Zhu, Jinjie Li, Zichao Li, Caiyan Chen, Mi Ma, Hongliang Zhang, Zhenyan He

**Affiliations:** 1 Key Laboratory of Plant Resources, Institute of Botany, Chinese Academy of Sciences, Beijing, 100093 China; 2 Key Lab of Crop Heterosis and Utilization of Ministry of Education, Beijing Key Lab of Crop Genetic Improvement, China Agricultural University, Beijing, 100193 China; 3 College of Biological Sciences and Biotechnology, Beijing Forestry University, Beijing, 100083 China; 4 University of Chinese Academy of Sciences, Beijing, 100049 China; 5 Key Laboratory of Agro-Ecological Processes in Subtropical Region, Institute of Subtropical Agriculture, Chinese Academy of Sciences, Changsha, 410125 China

**Keywords:** Plant breeding, Abiotic, Agricultural genetics, Genome-wide association studies

Correction to: *Nature Communications* 10.1038/s41467-019-10544-y, published online 12 June 2019.

The original version of this Article did not acknowledge Jianyin Xie as an equally contributing author with a superscript 6. Also, there was an error in the second sentence of the Abstract, which incorrectly read ‘While several transport systems have been reported, the complicity of rice Cd transport and accumulation indicates the necessity of identifying additional genes, especially those that are responsible for Cd accumulation divergence between *indica* and *japonica* rice subspecies.’ The correct version states ‘complexity’ in place of ‘complicity’. This has now been corrected in both the PDF and HTML versions of the Article.

