# Supplementary material for: Variation of a major facilitator superfamily gene contributes to differential cadmium accumulation between rice subspecies
Source: Nat Commun. 2019 Jun 12;10:2562. doi: 10.1038/s41467-019-10544-y (PMC6561962; doi:10.1038/s41467-019-10544-y)
Supplement: Supplementary file 1 — Supplementary Information [file 41467_2019_10544_MOESM1_ESM.pdf]

Variation of a major facilitator superfamily gene contributes to differential cadmium accumulation between rice subspecies

Yan *et al.*

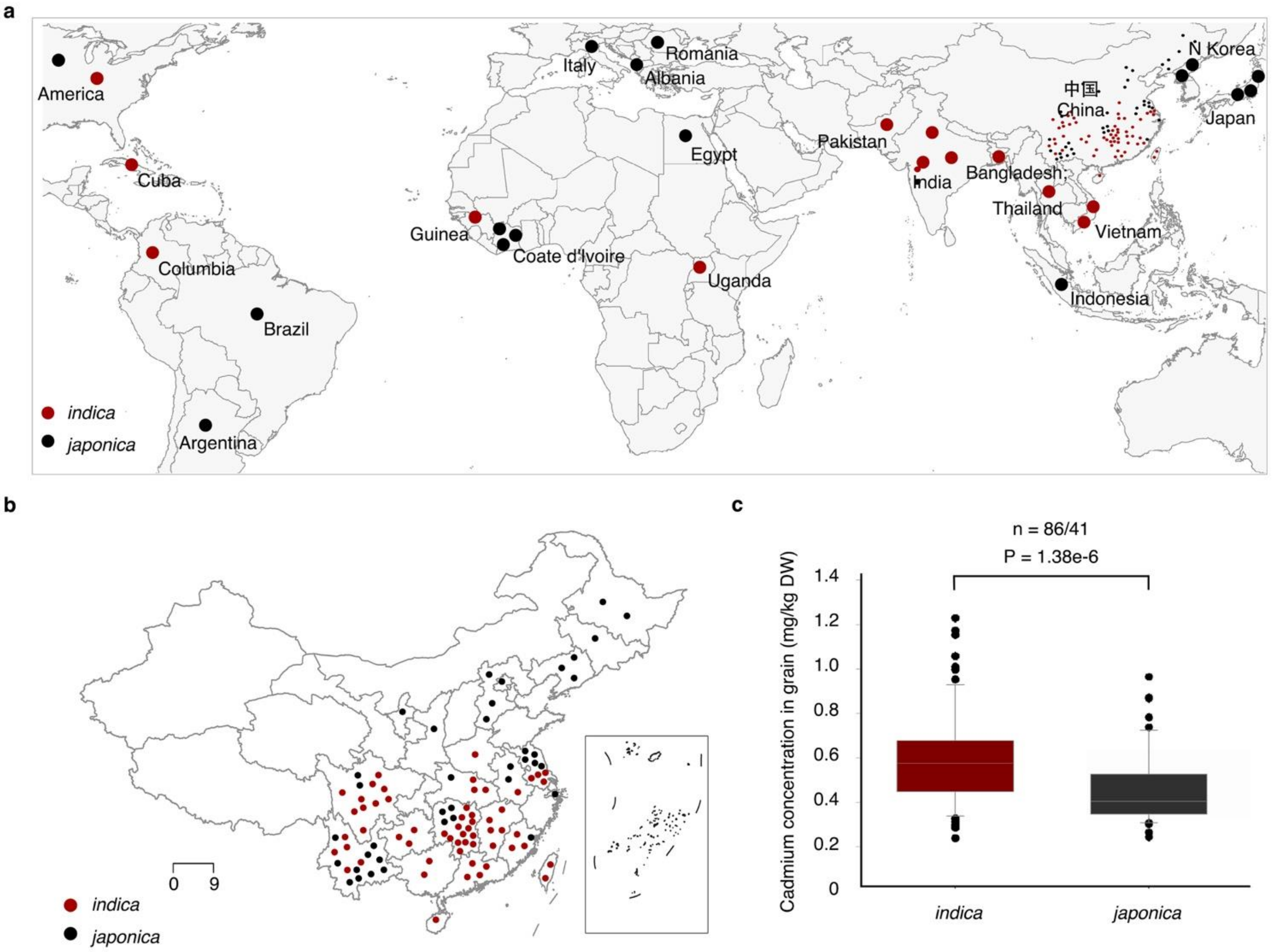

Supplementary Figure 1. *Indica* varieties show higher grain Cd accumulation level than *japonica* varieties.

(a) and **(b)** The geographical distribution of rice cultivars in the world **(a)** and China **(b)**. Red dots indicate the *indica* cultivars and black dots indicate *japonica* cultivars. **(c)** Comparison of grain Cd concentration between 86 *indica* (red) and 41 *japonica* (black) varieties. In each box plot, the bold horizontal line indicates the median, the edges of the box represent the first and third quartiles, and whiskers extend to span a 1.5 interquartile range from the edges. Data were designed with three replications and statistical comparison was performed by one-side t-test. Source data of Supplementary Figure 1c are provided as a Source Data file.

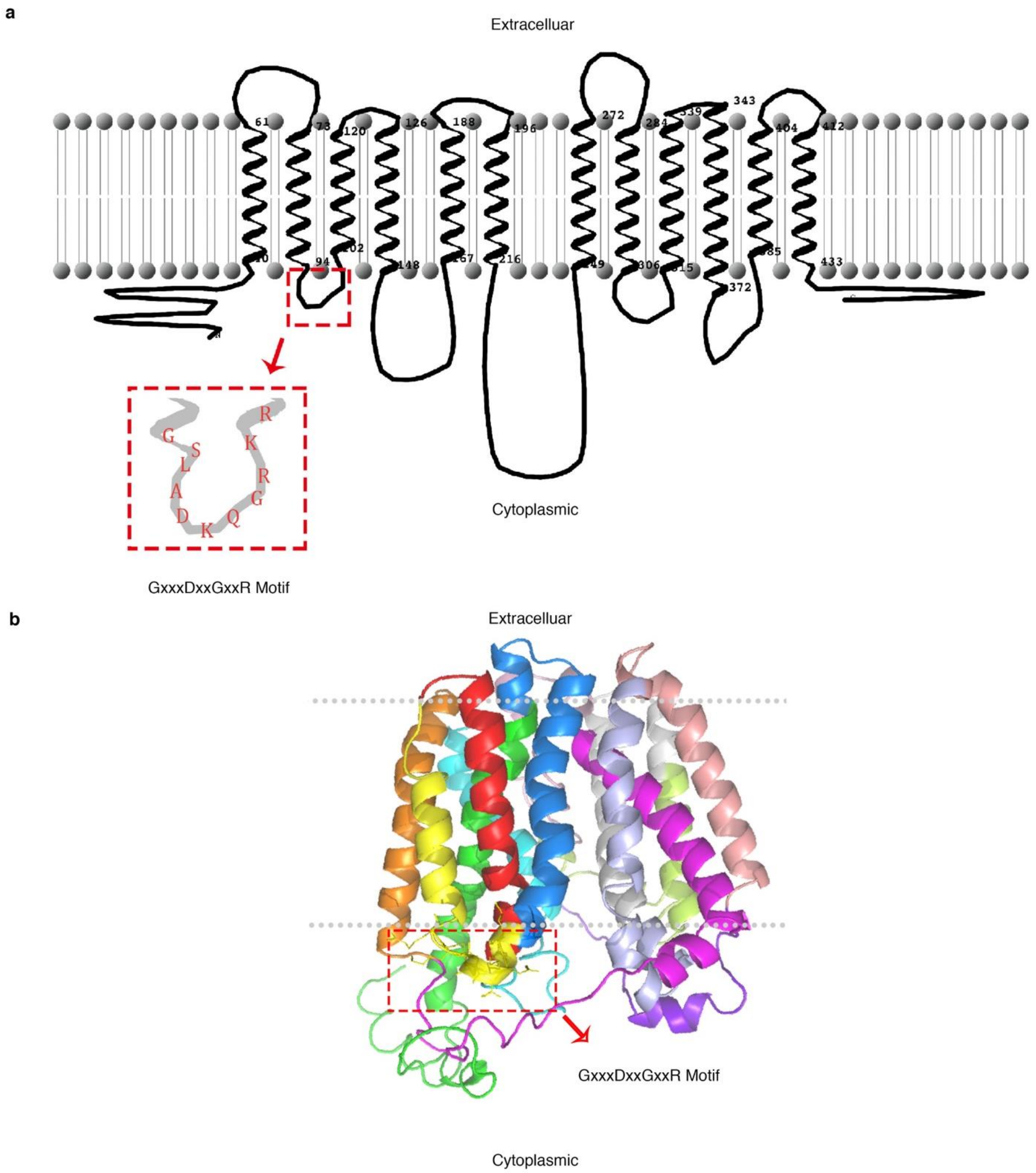

Supplementary Figure 2. Secondary and tertiary analysis of OsCd1.

(a) Secondary structure of OsCd1 protein, predicted by using Phyre2 and showed by TMRPres2D. Conserved motif is indicated in the red box. **(b)** The 3D model of OsCd1, predicted by using Phyre2 and showed by PyMOL. Conserved motif is indicated in the red box.

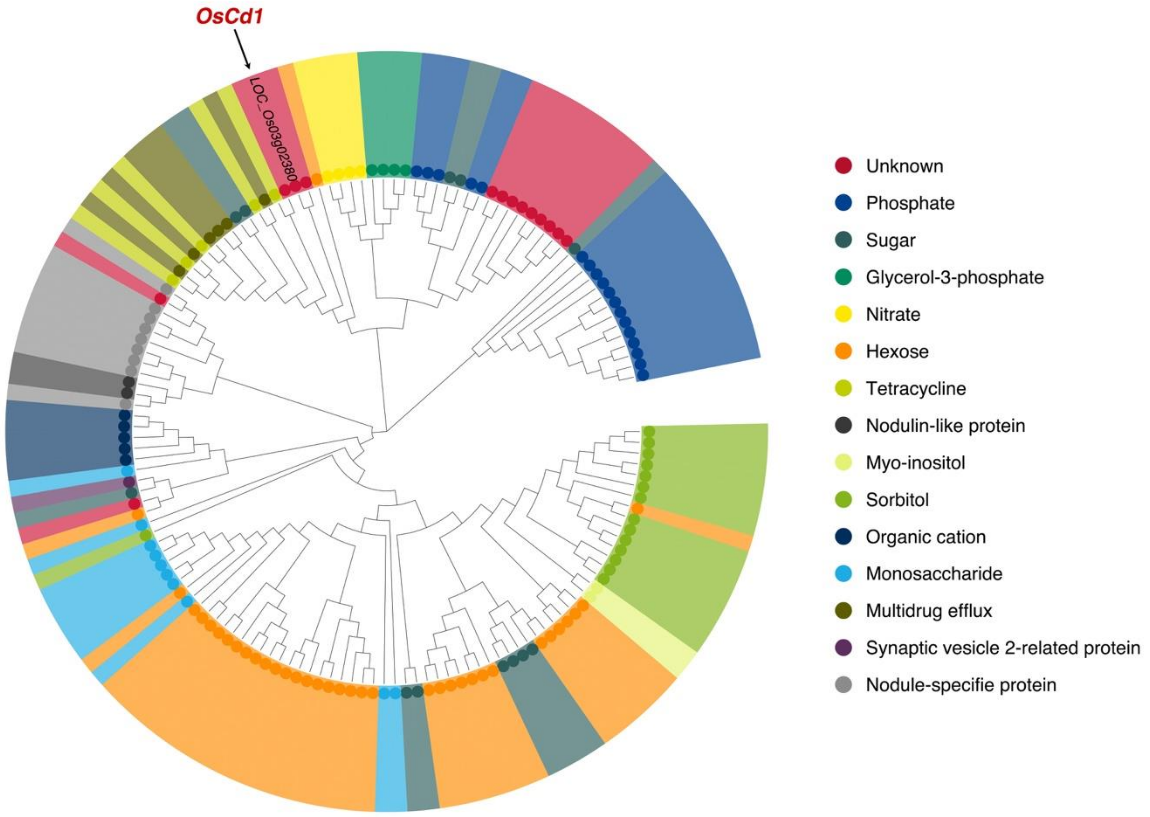

Supplementary Figure 3. Phylogenic analysis of *OsCd1* with other MFS members in rice.

Neighbor-joining (NJ) phylogenetic relationship of *OsCd1* to other MFS members in rice. *OsCd1* is marked, MFS proteins with different predicted substrates are indicated with different colors.

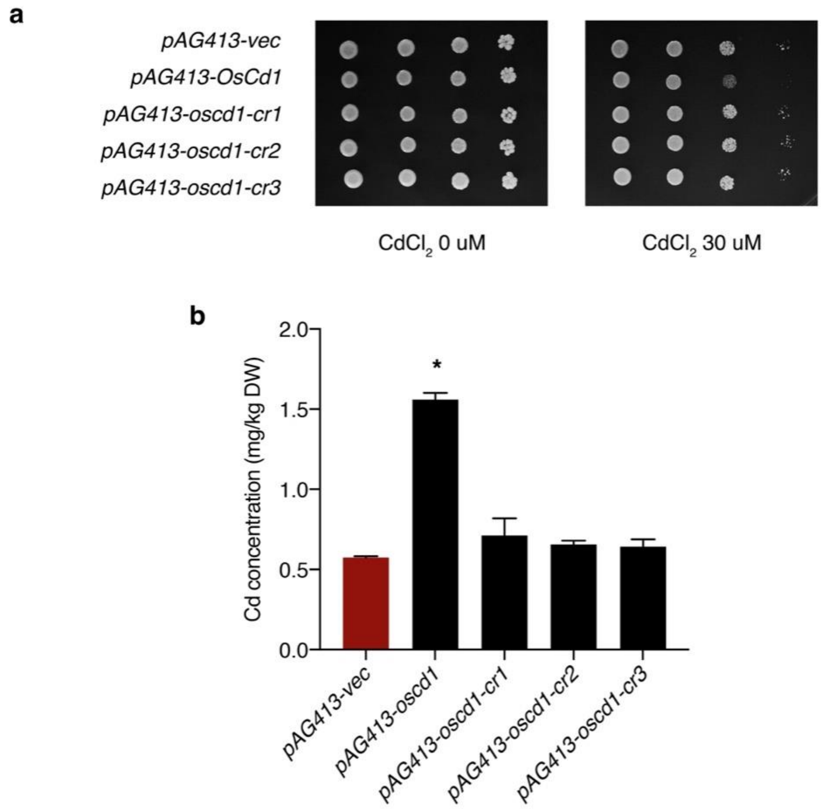

Supplementary Figure 4. Function characterization of the *OsCd1 CRISPR/Cas9* mutagenesis.

(a) Dilution-series spot assays of yeast treated with and without 30 μM $CdCl_2$. **(b)** Cd accumulation in wild type yeast W303 (red) and W303 expressing *oscd1CR-1*, *oscd1CR-2* and *oscd1CR-3* (black) treated with $CdCl_2$ for 24h. Error bars indicate standard deviation. Data from the transgenic lines were designed with three replications and statistical comparison was performed by one-side t-test (*P<0.05). Source data of Supplementary Figure 4b are provided as a Source Data file.

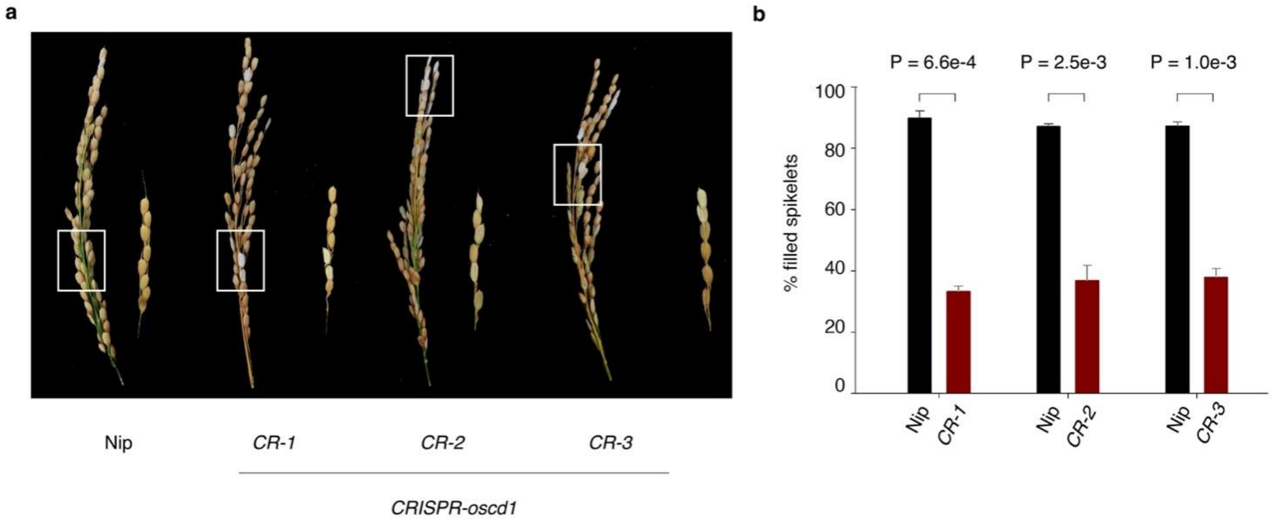

Supplementary Figure 5. Effect of *OsCd1* mutation on rice productivity.

(a) Growth of the spikelet in wild-type rice and three*CRIPSR-oscd1* lines at harvest. **(b)** Fertility of the seeds. The *CRIPSR-oscd1* lines were shown in red and *Nipponbare* in black. Error bars indicate standard deviation. Data from the transgenic lines were designed with at least five replications and statistical comparison was performed by one-side t-test. All data were compared with *Nipponbare*. Source data of Supplementary Figure 5b are provided as a Source Data file.

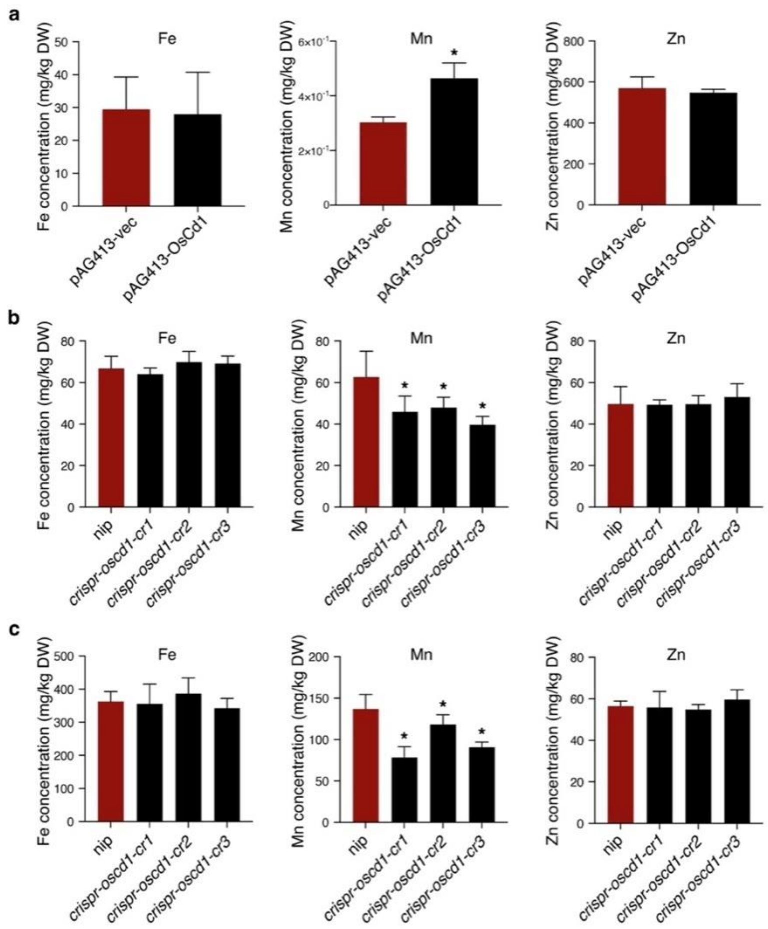

Supplementary Figure 6. Fe, Mn, Zn concentration in the *OsCd1* transgenic yeast, and the root and shoot in *crispr-oscd1* lines.

(**a**) Fe, Mn, Zn accumulation in wild type yeast W303 (red) and W303 expressing OsCd1 (Black) for 24h. **(b-c)** Concentration of Fe, Mn, Zn in the root **(b)** and shoot **(c)**. The *CRISPR-oscd1* lines were shown in black and the wild-type line was shown in red. Error bars indicate standard deviation. Data from the transgenic lines were designed with at least three replications and statistical comparison was performed by one-side t-test (*P<0.05). Source data of Supplementary Figure 6a-c are provided as a Source Data file.

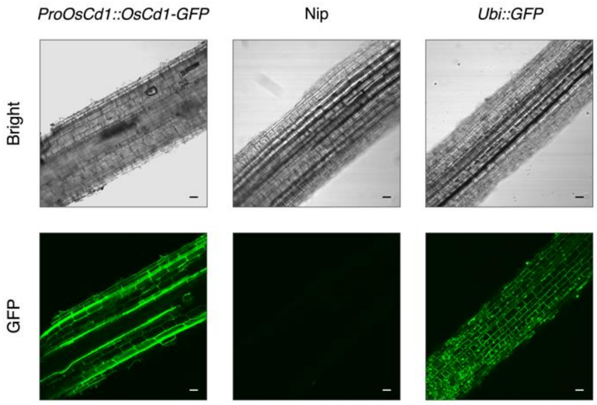

Supplementary Figure 7. The tissue expression of *OsCd1* in longitudinal frozen sections.

The GFP fluorescence of *proOsCd1*::*OsCd1*-GFP, NIP (negative control) and Ubi::GFP (positive control) observed by confocal laser scanning microscopy. Bars = 10 μm.

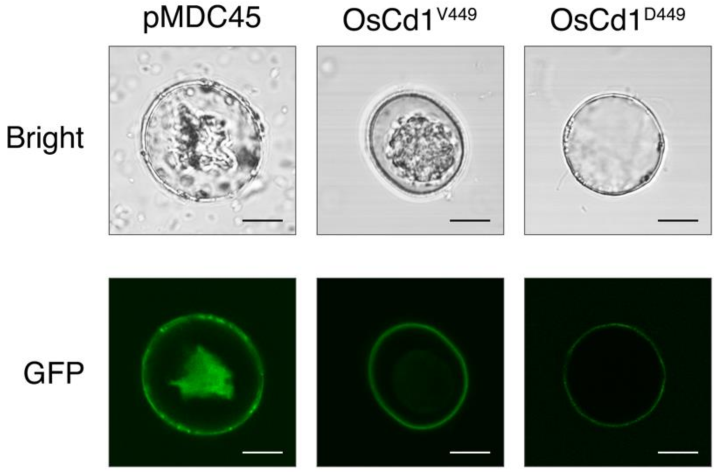

Supplementary Figure 8. Subcellular location of *OsCd1* in root protoplasts.

The GFP fluorescence of pMDC45-OsCd1^{V449} and pMDC45-OsCd1^{D449} observed by confocal laser scanning microscopy. Bars=10 μm.

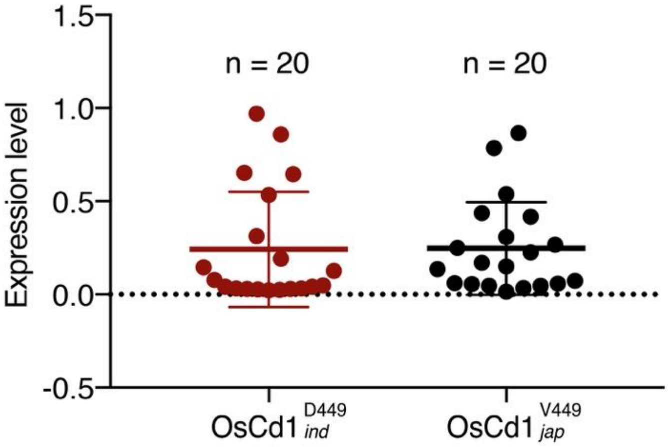

Supplementary Figure 9. Expression level of *OsCd1* in 20 *indica* and 20 *japonica* cultivars.

The expression level of *OsCd1* was detected in 20 *indica* (red) and 20 *japonica* (black) cultivars using qPCR. Data were designed with three replications. Source data are provided as a Source Data file.

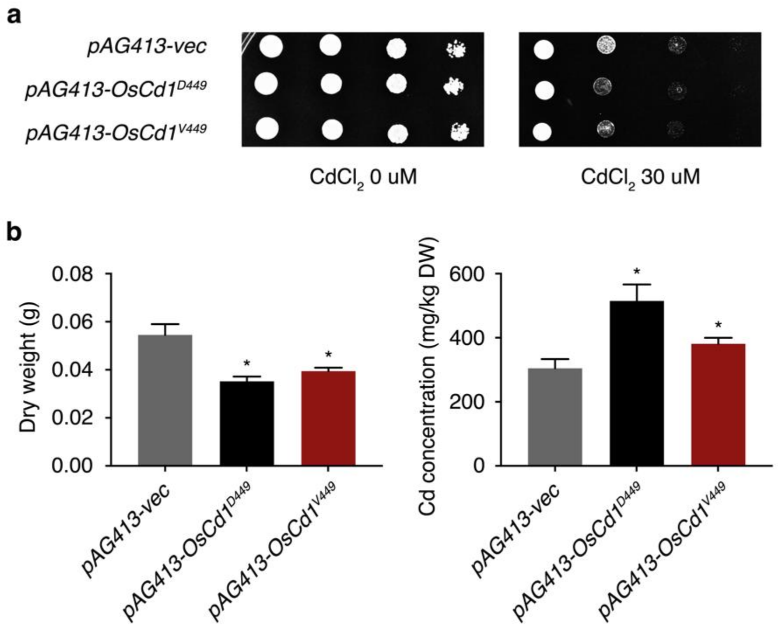

Supplementary Figure 10. Cd transport activity of OsCd1^{V449} and OsCd1^{D449} in *Δycf1* yeast mutant.

(a) Dilution-series spot assays of yeast treated with and without 30 μM $CdCl_2$. **(b)** Dry weight of yeast mutant *Δycf1* (grey) and *Δycf1* expressing OsCd1^{V449} (red) and OsCd1^{D449} (black) treated with $CdCl_2$ for 24h. **(c)** Cd accumulation in yeast mutant *Δycf1* (grey) and *Δycf1* expressing OsCd1^{V449} (red) and OsCd1^{D449} (black) treated with $CdCl_2$ for 24h. Error bars indicate standard deviation. Data from the transgenic lines were designed with three replications and statistical comparison was performed by one-side t-test (*P<0.05). Source data of Supplementary Figure 10b and 10c are provided as a Source Data file.

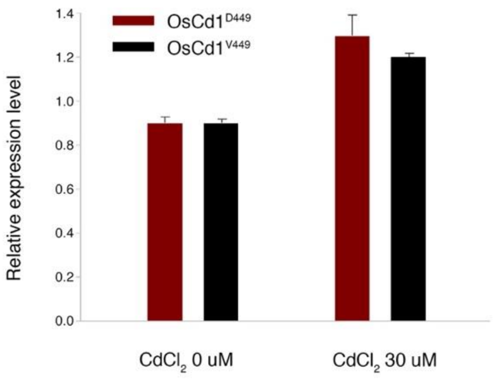

Supplementary Figure 11. Expression level of*OsCd1* in transgenic yeast.

Expression level of OsCd1[D449] (red) and OsCd1[V449] (black) in transgenic yeast were detected qPCR. Data were designed with three replications. Source data are provided as a Source Data file.

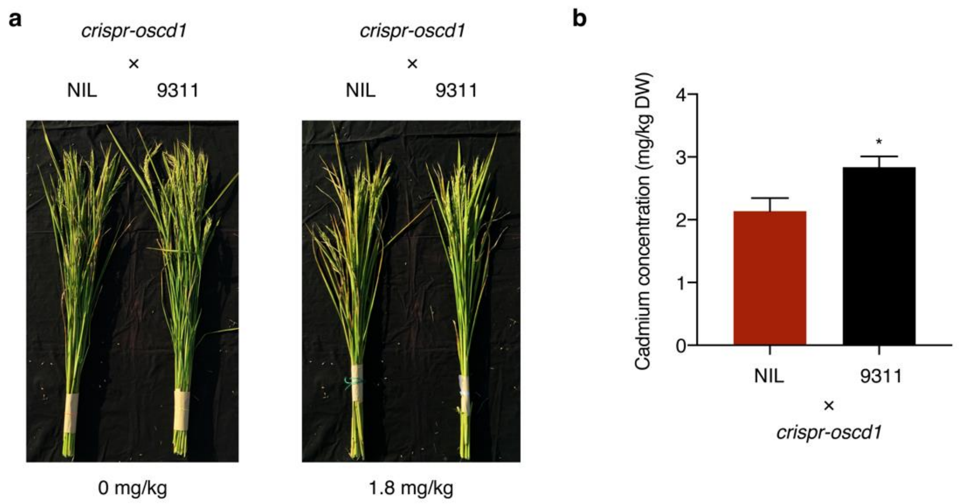

Supplementary Figure 12. The grain Cd accumulation of F1s by crossing NILs and 9311 to the *CRISPR-oscd1* knockout lines.

(a) Growth of F1s after treated with 1.8 mg/kg Cd at harvest. **(b)** Concentration of Cd in the brown rice. Error bars indicate standard deviation. Statistical analysis was inspected using one-side t-test. Data from the transgenic lines were designed with at least five replications (*$P<0.05$). Source data of Supplementary Figure 12b are provided as a Source Data file.

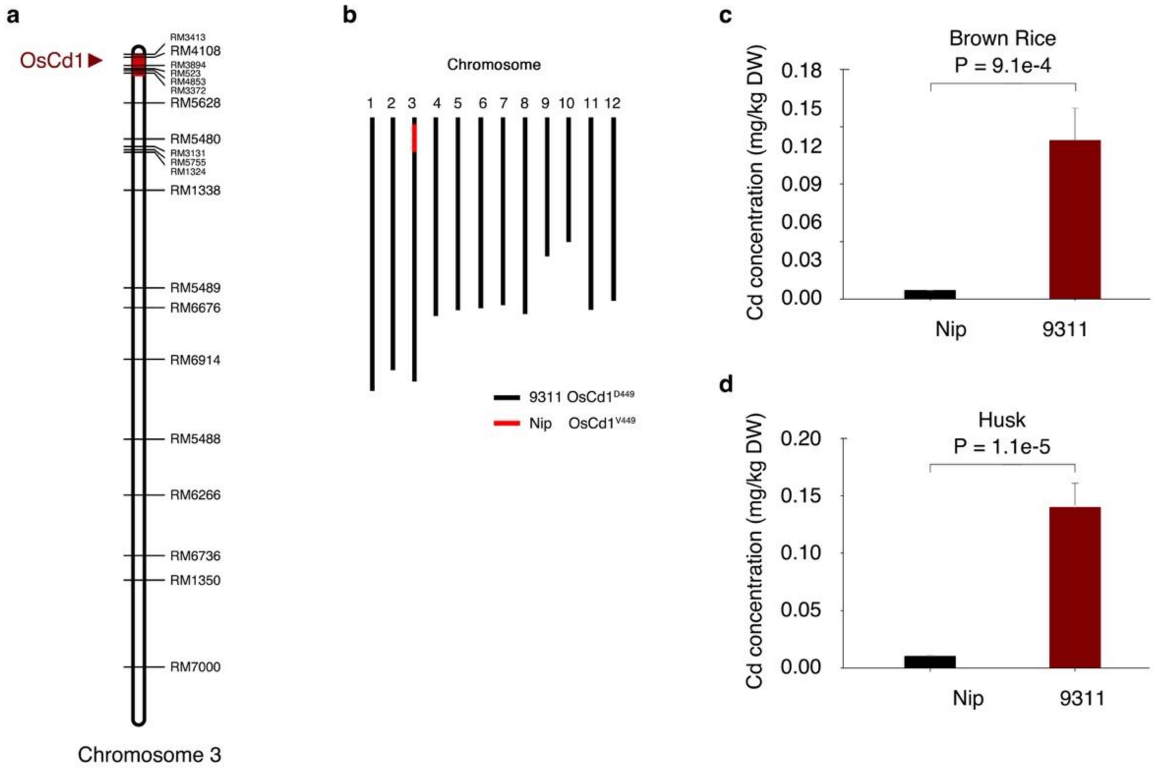

Supplementary Figure 13. Chromosome map of NIL genotype and grain Cd accumulation in parental 9311 and *Nipponbare*.

(a) NIL genotype of *OsCd1* on chromosome 3. Red bar represents the substituted segments in NIL. Dashes indicate the simple sequence repeats (SSR) between *Nipponbare* and 9311. **(b)** NIL genotype of *OsCd1* among chromosomes. Black bar, genomic region from 9311; red bar, genomic region from *Nipponbare*. **(c and d)** Cd concentration in the brown rice (**c**) and husk (**d**). Cd concentration of 9311 is shown in red and *Nipponbare* in black. Error bars indicate standard deviation. Data from the transgenic lines were designed with three replications and statistical comparison was performed by one-side t-test. Source data of Supplementary Figure 13c and 13d are provided as a Source Data file.

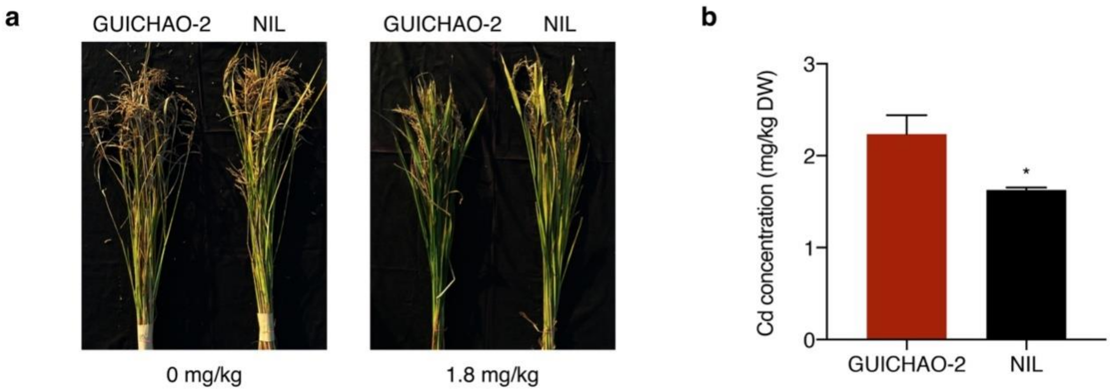

Supplementary Figure 14. Grain Cd accumulation of OsCd1^{V449} introgression lines of GUICHAO-2 background.

(a) Growth of GUICHAO-2 and NIL at harvest after 1.8 mg/kg Cd treatment in the field. **(b)** Cd concentration in brown rice between GUICHAO-2 (red) and NIL (black). Error bars indicate standard deviation. Data from the transgenic lines were designed with at least five replications and statistical comparison was performed by one-side t-test (*$P<0.05$). Source data of Supplementary Figure 14b are provided as a Source Data file.

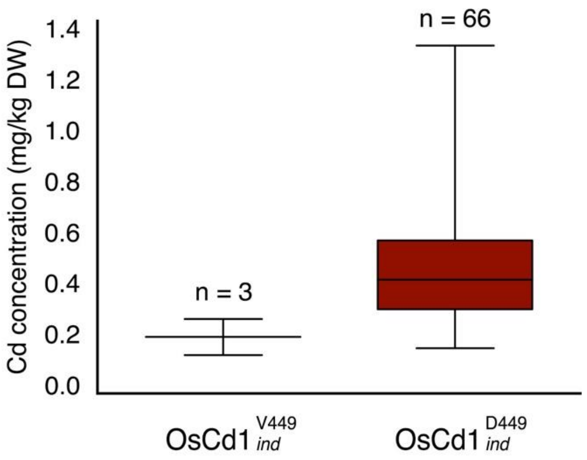

Supplementary Figure 15. Grain Cd accumulation of *indica* rice cultivars with different *OsCd1* genotypes.

Grain Cd accumulation of $OsCd1^{V449}$ and $OsCd1^{D449}$ in *indica* are displayed by the box plot; n denotes the number of genotypes belonging to each group. In each box plot, the bold horizontal line indicates the median, the edges of the box represent the first and third quartiles, and whiskers extend to span a 1.5 interquartile range from the edges. Source data are provided as a Source Data file.

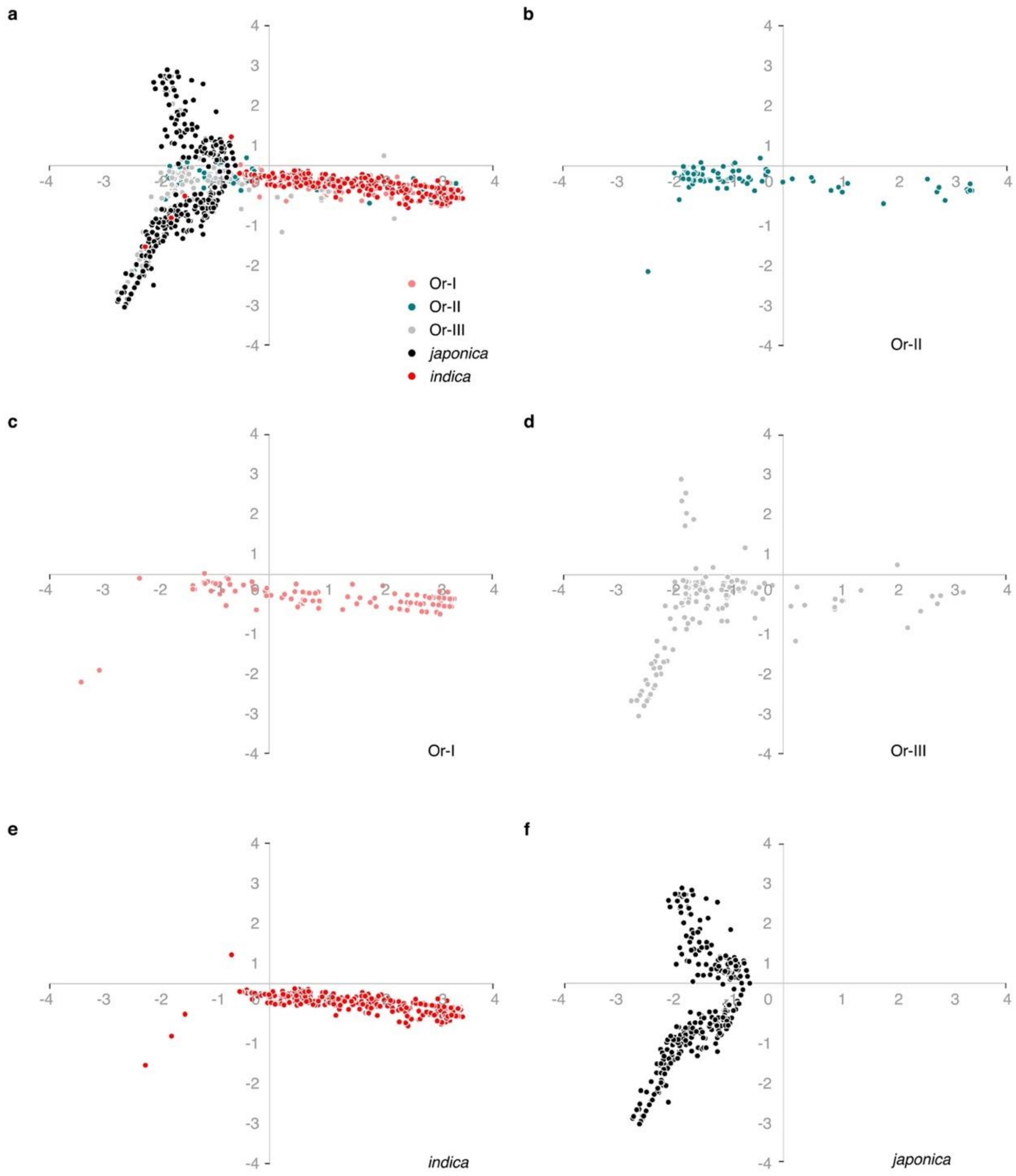

Supplementary Figure 16. PCA plots of the first two components of a full population (446 *O. rufipogon* accessions and 950*O. sativa* varieties).

Three types of *O. rufipogon* (Or-I **(c)**, Or-II **(b)** and Or-III **(d)**) were colored in pink, light blue and light grey. Two subspecies of *O. sativa* (*indica* **(e)** and *japonica* **(f)**) were colored in black and red, respectively.

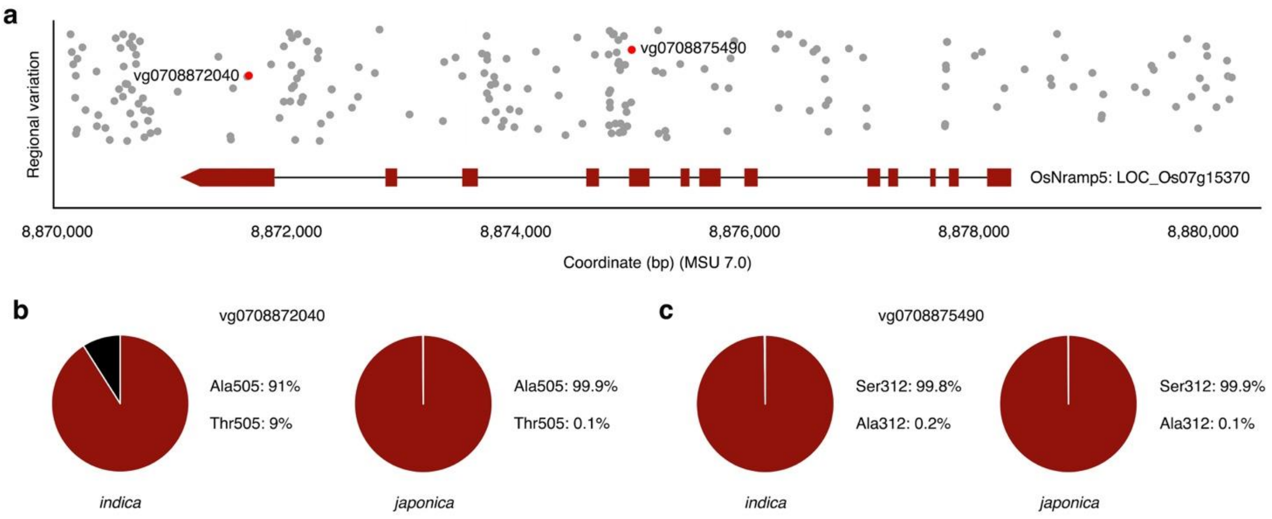

Supplementary Figure 17. Genotypes of OsNRAMP5 among rice cultivars.

(**a**) Gene structure and SNPs of OsNRAMP5. Each dot indicates a SNP. Causative SNPs were indicated in red. **(b)** and **(c)** Allele frequencies of causative SNPs (vg0708872040 and vg0708875490) in *japonica* and *indica*.

Supplementary Table 1. QTLs identified from genome-wide association study (GWAS) of rice grain Cd accumulation.

Name	Chromosome	start	stop	Contribution
QTL1	chr2	6756894	7048723	17.0%
QTL2	chr2	8915797	8951020	16.3%
QTL3	chr3	519263	995454	20.7%
QTL4	chr3	1222782	1321781	16.1%
QTL5	chr3	1681658	1907570	15.1%
QTL6	chr4	27363626	27685989	17.1%
QTL7	chr5	9283326	9577230	15.8%
QTL8	chr5	9889823	10112084	17.8%
QTL9	chr5	12006893	12200901	14.1%
QTL10	chr6	175373	304839	16.6%
QTL11	chr10	20003546	20361883	20.7%
QTL12	chr10	20363169	20860856	17.1%

Supplementary Table 2. The SNP22 among 127 rice cultivars.

SNP22	*indica*	*japonica*
**AA**	85	4
**AT**	1	2
**TT**	3	36

Supplementary Table 3. The SNP22 among 950 rice cultivars and 446 wild rice species.

Population	Frequency of base A	Frequency of base T
japonica	0%	100%
indica	99%	1%
**Or-I**	94.60%	5.40%
**Or-II**	22%	78%
**Or-III**	8.40%	91.60%

Supplementary Table 4. Sequences of primers used in this study.

Name	Sequence
OsCd1 S	ATGGAGGTGTTCTACTACCTCGTG
OsCd1 R	TTAAGGATTCAGTGGCTCATCTTCATCATC
pAG413-OsCd1 S	gaaaaaaccccggattctagaATGGAGGTGTTCTACTACCTCGTG
pAG413-OsCd1 R	taactaattacatgactcgagTTAAGGATTCAGTGGCTCATCTTCATCATC
pMDC45-OsCd1 S	gtaaaacgacggccagtgccGTGCAGCGTGACCCGGTCGTGCCCC
pMDC45-OsCd1 R	actcattttttctaccggtaTTGAAGCGGAGGTGCCGACGGGTGG
pCAMBIA1391Z-OsCd1 S	cccaagcttTACAGGTCATTCAACCTCACAGCCT
pCAMBIA1391Z-OsCd1 R	ccggaattcTTGGCGGCGTTACTGACACAATTCT
qPCR-OsCd1 S	TCAGCTGCATCACCAAGCACT
qPCR-OsCd1 R	TCTCTTGTTGTGCTCCGCGA
qPCR-Histone S	AGTTTGGTCGCTCTCGATTTCG
qPCR-Histone R	TCAACAAGTTGACCACGTCACG
CRISPR-OsCd1-CR1 S	CCTGTGAGGCTGTGAGTCTG
CRISPR-OsCd1-CR1 R	TGCAACCTTGAACTGGGACA
CRISPR-OsCd1-CR2 S	CTGTGTGTTTCAGGGGGAGG
CRISPR-OsCd1-CR2 R	AAAGCATCAGTGTGAGGGGG
CRISPR-OsCd1-CR3 S	CTTGGCGCATTTTCGTCCTC
CRISPR-OsCd1-CR3 R	GAAATGTGTGAGCGTCCAGT

Supplementary Table 5. SgRNA in *CRISPR-oscd1* vector construction.

Name	sgRNA
CRISPR-oscd1 CR-1	GCCTGGTCGCAATTGTATCC
CRISPR-oscd1 CR-2	GCTTCTCGGCGTTCGAGTCA
CRISPR-oscd1 CR-3	GATGAGGATCTTGTACTCGG
