# Supplementary material for: Variation of a major facilitator superfamily gene contributes to differential cadmium accumulation between rice subspecies
Source: Nat Commun. 2019 Jun 12;10:2562. doi: 10.1038/s41467-019-10544-y (PMC6561962; doi:10.1038/s41467-019-10544-y)
Supplement: Supplementary file 3 — Description of Additional Supplementary Files [file 41467_2019_10544_MOESM3_ESM.docx]

**Description of Additional Supplementary Files**

File Name: Supplementary Data 1

Description: Grain cadmium accumulation in *japonica* and *indica* cultivars

File Name: Supplementary Data 2
Description: Annotated genes among the 12 QTLs.

File Name: Supplementary Data 3
Description: The 13 candidate genes and the GoSlim assignments for annotated genes in QTLs

File Name: Supplementary Data 4
Description: Members of Major Facilitator Superfamily (MFS) in rice

File Name: Supplementary Data 5
Description: OsCd1 sequence variation in 127 rice cultivars
